# Histone deacetylase inhibitor resminostat in combination with sorafenib counteracts platelet-mediated pro-tumoral effects in hepatocellular carcinoma

**DOI:** 10.1038/s41598-021-88983-1

**Published:** 2021-05-05

**Authors:** Gundula Streubel, Sabine Schrepfer, Hannah Kallus, Ulrike Parnitzke, Tanja Wulff, Frank Hermann, Matthias Borgmann, Svetlana Hamm

**Affiliations:** 4SC AG, Fraunhoferstraße 22, 82152 Planegg-Martinsried, Germany

**Keywords:** Cancer microenvironment, Tumour biomarkers, Liver cancer, Predictive markers, Drug development, Acetylation, Phosphorylation, Cell invasion, Cell growth, Mechanisms of disease

## Abstract

In hepatocellular carcinoma (HCC), blood platelets have been linked to tumor growth, epithelial-to-mesenchymal transition (EMT), extrahepatic metastasis and a limited therapeutic response to the multikinase inhibitor (MKi) sorafenib, the standard of care in advanced HCC for the last decade. Recent clinical data indicated an improved overall survival for sorafenib in combination with the HDAC inhibitor resminostat in a platelet count dependent manner. Here, the impact of platelets on the sorafenib and resminostat drug effects in HCC cells was explored. In contrast to sorafenib, resminostat triggered an anti-proliferative response in HCC cell lines independent of platelets. As previously described, platelets induced invasive capabilities of HCC cells, a prerequisite for extravasation and metastasis. Importantly, the resminostat/sorafenib drug combination, but not the individual drugs, effectively blocked platelet-induced HCC cell invasion. Exploration of the molecular mechanism revealed that the combined drug action led to a reduction of platelet-induced *CD44* expression and to the deregulation of several other epithelial and mesenchymal genes, suggesting interference with cell invasion via EMT. In addition, the drug combination decreased phosphorylated ERK level, indicating inhibition of the mitogenic signaling pathway MEK/ERK. Taken together, the resminostat plus sorafenib combination counteracts platelet-mediated cancer promoting effects in HCC cells.

## Introduction

Liver cancer is the second most frequent cause of cancer-related death and its increasing incidence constitutes a growing global health burden. Hepatocellular carcinoma (HCC) accounts for up to 90% of all primary liver cancers and is considered a heterogenous disease in terms of etiology and underlying genetic alterations. The disease is associated with a poor prognosis and in fact, < 10% of patients with HCC can be cured^1,2^. Highlighting the need for effective systemic therapies, sorafenib, a multikinase inhibitor (MKi) and the standard of care first-line therapy in advanced, unresectable HCC for the past decade, only moderately improves the median overall survival for several months^3,4^. Unfortunately, other recently approved kinase inhibitors, such as lenvatinib, regorafenib, and cabozantinib, fail to extend the overall survival for more than a year^5^. While this limited therapeutic success is being attributed to the existence of primary and acquired drug resistance mechanisms, it is becoming increasingly evident that an important limitation to the success of HCC therapies is the lack of specific biomarkers to predict treatment responses in patients^2,6^.

Recent findings indicate a role for blood platelets (thrombocytes) in HCC disease progression and moderate drug response. Clinical data from HCC patients have revealed a link between elevated platelet count or platelet to lymphocyte ratio (PLR) and worse prognosis, shorter overall survival, and higher risk of recurrence after resection. Moreover, a correlation between platelet count and an increased risk of extrahepatic metastasis and the metastatic potential of HCC cells has been observed^7–10^. Indeed, as constituents of the tumor microenvironment (TME) and of the blood, platelets have long been recognized as playing an important role in the development and progression of solid cancers^11^. They have been reported to promote tumor growth, cell proliferation, epithelial to mesenchymal transition (EMT), cancer metastasis and angiogenesis. Linking their function with tumor dissemination and metastasis, platelets were reported to enhance the escape of tumor cells from primary tumor sites by inducing cellular invasion. Moreover, platelets promote the survival of circulating cancer cells by protecting them against immune recognition and shear stress and support the formation of metastatic niches. One of the suggested mechanism of platelets is the release growth factors containing granules, which directly affect cancer cells and the surrounding tissue^7,12,13^. Importantly, emerging evidence also implicate platelets in drug resistance in several cancer indications^12–14^. Platelet-derived factors have been reported to antagonize the action of the multikinase inhibitors (MKi) sorafenib and regorafenib in HCC cell lines^15,16^. Taken together, the role of platelets might be critical for the development of therapeutic resistances and for the moderate clinical response of HCC patients.

Expanding on existing therapies, targeting histone deacetylase (HDAC) activities in HCC is of increasing interest^17^. Moreover, combining HDAC inhibitors (HDACi) with sorafenib has emerged as a potential treatment strategy in HCC^18–20^. Interestingly, a clinical trial (NCT02400788) conducted in East Asian patients with advanced HCC indicated that first-line treatment with the resminostat/sorafenib drug combination had a positive impact in patients with a normal-to-high platelet count at baseline^21^. As these findings suggested that platelet count could serve as a potential predictive factor for this therapy, we investigated how platelets modulate HCC cell features and the drug response to resminostat, sorafenib, and the drug combination.

## Results

### Inhibition of cancer cell growth by the broad-spectrum HDACi resminostat

The orally available hydroxamate HDACi resminostat increases histone lysine acetylation levels in cancer cells and patient blood samples^20,22^. To precisely determine the inhibitory activity of resminostat, we performed in vitro HDAC activity assays against the 11 human Zn^2+^-dependent HDAC proteins. Calculation of the half-maximal inhibitory concentration (IC50) revealed that resminostat inhibits the activity of several HDACs: Class I HDAC1, 2, 3, and 8; class II b HDAC6 and 10; and class IV HDAC11. In contrast, the class IIa HDACs were not inhibited by resminostat at pharmacologically relevant concentrations, with high IC50 values between 20 and 70 µM (Fig. S1A). These data reveal resminostat is a broad-spectrum HDAC inhibitor targeting class I, IIb and class IV HDACs. The HDACs inhibited by resminostat are known to have chromatin-directed, as well as cytosolic activities. As expected, resminostat caused an increase of the global acetylation level in HCC cells in a dose-dependent manner (Fig. S1B). Moreover, hyperacetylation of the specific HDAC substrates histone mark H3K27 and the non-histone protein α-Tubulin was detected upon resminostat treatment (Fig. S1B,C).

Cytotoxic and anti-proliferative effects of resminostat have been reported for several cancer cell types including HCC, multiple myeloma, and head and neck squamous cell carcinoma^22–24^. Indicating an anti-proliferative effect via cell cycle inhibition, resminostat treatment of HCC cells led to accumulation of the cell cycle inhibitor p21 protein (cyclin-dependent kinase inhibitor 1), an established HDAC target (Fig. S1C)^25,26^. Corroborating these findings, in a cell proliferation screen using a set of 74 cancer cell lines representing 14 different cancer types, resminostat inhibited cell growth in most of the tested cell lines across all tested indications (Fig. S1D and Table S1).

### Anti-tumoral effects of HDACi resminostat in combination with the MKi sorafenib in an orthotopic xenograft mouse model

**Figure 1:**
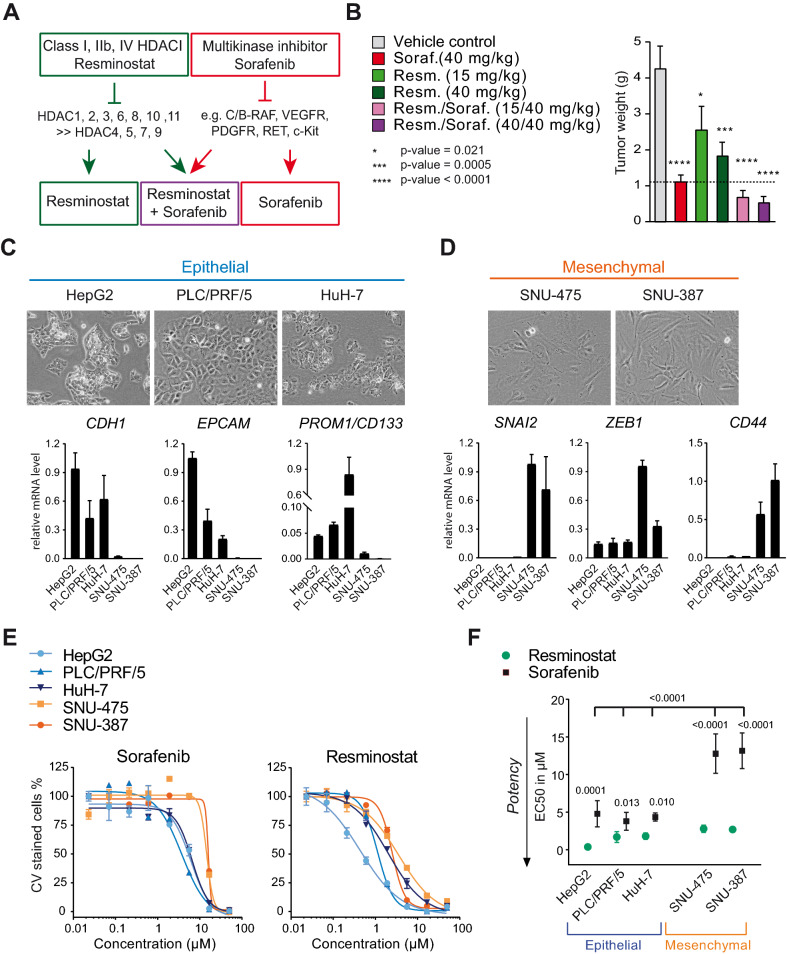
Resminostat exhibits anti-proliferative activity in HCC cells with reduced sorafenib sensitivity. (**A**) Outline of the inhibitory profiles of the histone deacetylase inhibitor (HDACi) resminostat and the multikinase inhibitor (MKi) sorafenib. (**B**) Orthotopic xenograft mouse model of Hep3B2.1-7 human liver cancer cells in SCID mice. The weight of liver tumors from mice treated with resminostat, sorafenib, the drug combination or vehicle control was determined 21 days following the beginning of treatment (n = 10–12 mice per group). Data are presented as mean + SEM. For statistical analysis, one-way ANOVA and multiple comparison (Dunnett) was performed for compound treatments compared to vehicle control. The adjusted p-values with 95% confidence interval were reported as significant. (**C**) HCC cell lines with epithelial (blue) features as characterized by cell morphology and marker gene expression. A representative microscopic image for each cell line is shown (upper panels). The relative mRNA levels of biological replicates are presented as data means with SD (lower panels). (**D**) As in (**C**), but with HCC cell lines characterized as mesenchymal (orange). (**E**) Dose-response curves from proliferation assays with sorafenib and resminostat in epithelial and mesenchymal HCC cells. The drug concentration ranged from 0.02 to 50 µM. One representative experiment is shown. Data of three technical replicates are presented as mean ± SD. (**F**) EC50 values for resminostat and sorafenib in epithelial (HepG2, PLC/PRF/5 and HuH-7) and mesenchymal (SNU-475 and SNU-387) HCC cells. The data of biological replicates are presented as mean ± SD (n ≥ 3). One-way ANOVA with multiple comparison testing (Tukey) was performed. Multiplicity adjusted p-values for the EC50 with sorafenib versus resminostat for each cell line and comparison between cell lines for sorafenib are presented (95% confidence interval).

Sorafenib is a multikinase inhibitor (MKi) targeting serine/threonine RAF kinases, as well as several growth factor receptor tyrosine kinases^27^ (Fig. 1A). Combined sorafenib and resminostat drug activities have been previously described in HCC cell lines in vitro^23^. This prompted us to determine the in vivo anti-tumor activity of resminostat and sorafenib alone and in combination in the Hep3B.1-7 orthotopic xenograft mouse model for liver cancer^28^. Daily administration of resminostat (15 mg/kg and 40 mg/kg) and sorafenib (40 mg/kg) as mono-treatments significantly reduced tumor weight compared to the vehicle control (p-values depicted in Fig. 1B). The anti-tumoral effect was further increased when a combination of both drugs was administered (Fig. 1B).

### Resminostat exhibits anti-proliferative activity in HCC cells with reduced sorafenib sensitivity

Neoplastic HCC cells harbor heterogenous features of epithelial or mesenchymal cell types and several reports have suggested that the acquisition of mesenchymal features in HCC is involved in disease progression as well as in mediating resistance to sorafenib^29,30^. Moreover, a role of platelets in triggering EMT has been indicated for several cancer indications^8,12,13^.

Therefore, we compared epithelial (Fig. 1C) or mesenchymal (Fig. 1D) phenotypical features and marker gene expression of five HCC cell lines by microscopy and RT-PCR next. Epithelial cells are polygonal, have a cobblestone morphology, and grow as discrete colonies through the formation of tight cell to cell contacts. In contrast, mesenchymal or fibroblast-like cells have a spindle-shaped morphology, do not form tight cell-to-cell contacts and, therefore, do not grow as well-defined colonies. Moreover, several genes are known to correspond with either a more epithelial or mesenchymal cell type, and thus serve as representative marker genes^31^. The expression of such genes corresponding to an epithelial-like or mesenchymal-like cell type has been previously described for various established HCC cell lines^30,32–34^. Comparison of cell morphology and expression of a representative set of markers for epithelial (*EPCAM, CDH1/E-Cadherin, PROM1/CD133*) and mesenchymal (*SNAI2, CD44, ZEB1*) cells revealed that HepG2, PLC/PRF/5, and HuH-7 cells harbor features of epithelial cells, while SNU-475 and SNU-387 cells are characterized by mesenchymal features (Fig. 1C,D), thus confirming previous findings.

To determine the drug efficacies in epithelial and mesenchymal HCC cells, proliferation assays with increasing concentrations of sorafenib and resminostat ranging from 0.02 to 50 µM were performed. Dose-response curves for sorafenib were shifted towards higher drug concentrations in mesenchymal-like SNU-475 and SNU-387 cells compared to epithelial HepG2, PLC/PRF/5 and HuH-7 cells, indicating that sorafenib was less effective in mesenchymal HCC cells (Fig. 1E). This was also reflected by the EC50 values for sorafenib, which were about three-fold higher in SNU-475 and SNU-387 cells compared to the epithelial cell lines (Fig. 1F). Importantly, the dose-response curves and EC50s for resminostat were comparable in both cell types. In conclusion, sorafenib was less potent in mesenchymal than in epithelial cells, whereas resminostat’s potency appeared to be independent of the epithelial or mesenchymal phenotype. Moreover, resminostat was more potent in inhibiting cell growth compared to sorafenib (p-values Fig. 1F).

### The efficacy of the resminostat plus sorafenib combination was determined by resminostat and was independent of platelet factors

**Figure 2:**
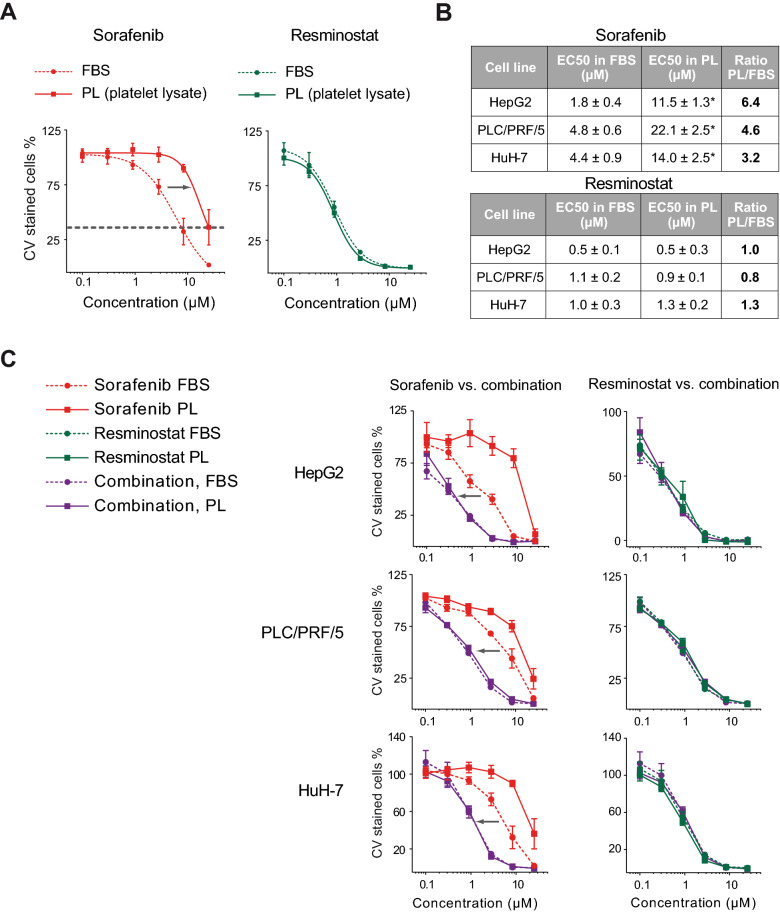
The efficacy of the resminostat plus sorafenib combination is determined by resminostat and is independent of platelet factors. (**A**) Proliferation assays in PLC/PRF/5 cells in the presence of fetal bovine serum (FBS) or platelet lysate (PL). The grey arrow indicates a shift in the sorafenib dose-response curve towards higher concentrations under the PL condition. Maximum inhibition at the highest concentration of 25 µM was not reached with PL (grey dotted line) compared to FBS. The data are presented as mean ± SD (n = 5). (**B**) EC50 data of sorafenib and resminostat in the presence of FBS or PL in three epithelial HCC cells lines. The absolute EC50 values (*) and the ratio of PL/FBS EC50s are shown. The data are presented as mean ± SD (n ≥ 4). One-way ANOVA with multiple comparison testing (Tukey) was performed. Multiplicity adjusted p-values with 95% confidence interval was applied: Differences in EC50 values for resminostat treatment with FBS compared to PL in HepG2, PLC/PRF/5 and HuH-7 were not significant. For sorafenib treatment with FBS compared to PL, the p-values were < 0.0001 in all three cell lines. (**C**) Proliferation assay in HepG2, PLC/PRF/5 and HuH-7 cells in the presence of FBS or PL. Dose-response curves from 0.1 to 25 µM comparing sorafenib mono-treatment with resminostat/sorafenib combination (left-side graphs) or resminostat mono-treatment with resminostat/sorafenib combination (right-side graph). The grey arrow indicates a difference in the potency of the drug combination compared to sorafenib. The data are presented as mean ± SD (n ≥ 4).

Platelet factors have been reported to antagonize the action of kinase inhibitors such as sorafenib^15^. Here, commercially available human platelet lysate (PL) derived from multiple platelet donors was used as a surrogate for the platelet releasates to investigate the impact of platelets on the efficacy of resminostat and sorafenib. Comparative proliferation assays in the presence of a platelet lysate or under standard growth conditions with fetal bovine serum (FBS) showed a shift of dose-response curves for sorafenib to higher sorafenib concentrations in the presence of platelet lysate. Moreover, the maximal inhibition was not reached at 25 µM. These results indicate that platelet factors reduced the sorafenib potency and efficacy (left-hand graph, Fig. 2A). In contrast, the potency and efficacy of resminostat was unaffected by platelet factors (right-hand graph, Fig. 2A). To further quantify this effect, the absolute EC50 values for sorafenib and resminostat mono-treatments in epithelial HepG2, PLC/PRF/5 and HuH-7 cells were determined. In all three cell lines, the EC50 values for sorafenib were three- to six-fold higher in the presence of platelet lysate, while the EC50 values for resminostat remained unchanged under both conditions (Fig. 2B). In the mesenchymal cell lines SNU-387 and SNU-475, which were already less sensitive to sorafenib, a minor shift in the dose-response curves for sorafenib was observed under platelet conditions, while resminostat’s anti-proliferative activity was unaffected (Fig. S2).

Then, proliferation assays comparing the single drug treatments with the drug combination were performed (Fig. 2C and S2). These revealed that the drug combination was more effective compared to sorafenib mono-treatment in the presence of platelet lysate and with FBS. Furthermore, a comparison of the dose curves for resminostat and the drug combination showed that resminostat determined the efficacy of the drug combination. Taken together, these data showed that resminostat was more potent and effective compared to sorafenib in HCC cells, even in the presence of platelet lysate, and that the antiproliferative effect of the resminostat/sorafenib drug combination was determined by resminostat regardless of platelet factors.

### Platelets induce cellular invasion of HCC cells

**Figure 3:**
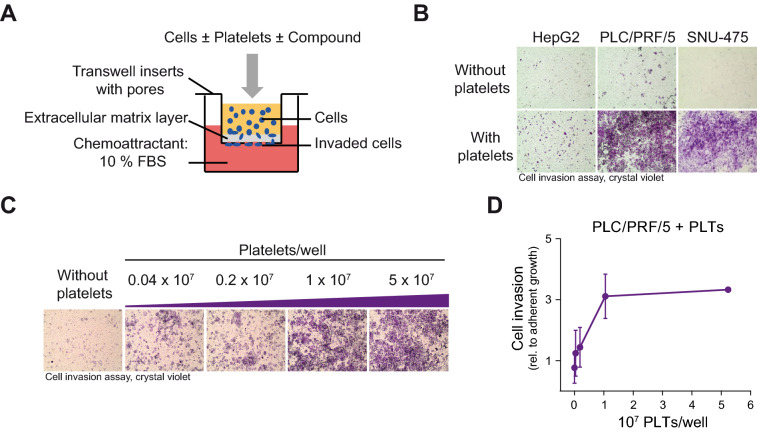
Platelets induce cellular invasion of HCC cells. (**A**) Schematic representation of the in vitro cell invasion assay in the presence of platelets. Platelets were isolated from human blood, combined with cells, and then placed into transwell inserts. FBS was used as a chemoattractant. Invaded cells at the bottom side of the transwells were stained with crystal violet for microscopy and photometrical quantification. (**B**) Cell invasion assay of HepG2, PLC/PRF/5 and SNU-475 cells with and without platelets (1.25 × 10^7^ PLTs/well). Microscopic images show invaded and crystal violet stained cells. (**C**) Cell invasion assay of PLC/PRF/5 cells with an increasing number of platelets, 0.04 × 10^7^ PLTs/well (1.6 × 10^3^ PLTs/mm^3^) up to 5 × 10^7^ PLTs/well (200 × 10^3^ PLTs/mm^3^). Microscopic images of invaded and crystal violet stained cells are shown. (**D**) Quantification of PLC/PRF/5 cell invasion assay with an increasing number of platelets. The OD values from invasion assays were normalized to ODs from adherent growth. Data are presented as mean ± SD (n = 3).

Cell invasion allows cancer cells to become motile, to navigate through the extracellular matrix of the neighboring tissue, and to subsequently intravasate and infiltrate distant organs. Using transwell invasion assays, we analyzed the invasive capacity of HCC cells. Cells that invade the matrigel extracellular matrix layer and migrate through the pores of the transwell can then be detected by crystal violet staining at the bottom side of the transwell insert (Fig. 3A).

In a previous report, the direct interaction between tumor cells and platelets was sufficient to induce cell invasion and to prime tumor cells for subsequent metastasis in a breast cancer cell model^35^. To investigate the influence of platelets on the invasive capacities of HCC cells, platelets were freshly isolated from human blood and used together with HepG2, PLC/PRF/5 and SNU-475 cells in transwell invasion assays. The addition of platelets enhanced the invasiveness of PLC/PRF/5 and SNU-475 cells, and slightly enhanced the invasiveness of HEPG2 cells (Fig. 3B). Other than platelets, treatment of HCC cells with platelet lysate did not induce cellular invasion, implying that a direct interaction of platelets or platelet debris with the cells is required for the induction of cell invasion (Fig. S3A). Furthermore, cell invasion was gradually induced with an increasing platelet number (Fig. 3C,D). Monitoring the adherent growth in the presence of platelets in parallel to the invasion assay ruled out the possibility that the observed induction of cell invasion could be explained by an augmented cell growth (Fig. S3B,C). As an additional control, platelets were seeded in transwell invasion chambers without cells. Background staining was not visible even with the highest used platelet numbers (Fig. S3D). In summary, blood platelets induced cellular invasion of HCC cells.

### Platelet-induced cell invasion is inhibited by the resminostat/sorafenib combination

**Figure 4:**
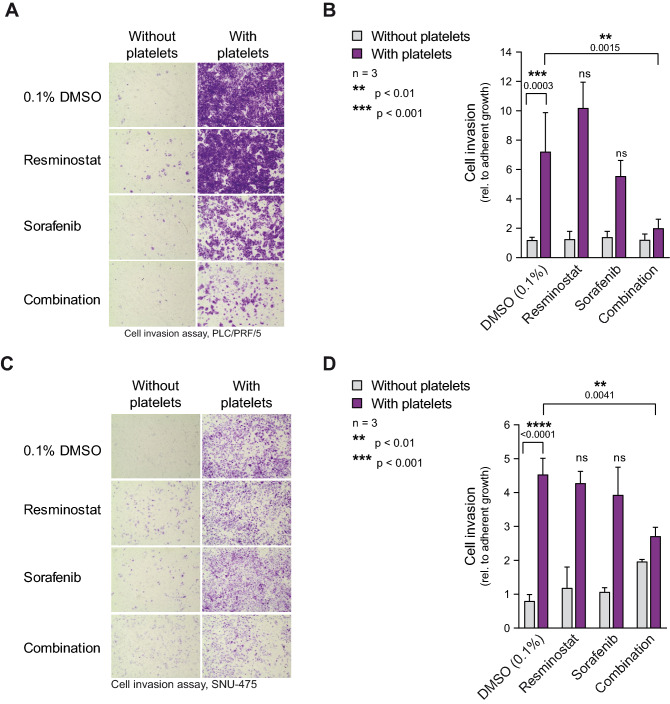
Platelet-induced cell invasion is reversed by resminostat in combination with sorafenib. (**A**) Effect of resminostat/sorafenib drug combination on platelet-induced cell invasion. PLC/PRF/5 cell invasion assay with 2.5 × 10^7^ PLTs/well (100 × 10^3^ PLTs/mm^3^) and without platelets. 1 µM resminostat and 2.5 µM sorafenib were used in mono- and combination treatments, 0.1% DMSO was used as the vehicle control. Representative microscopic images of transwells. (**B**) Quantification of cell invasion (right). The data are presented as mean ± SD (n = 3). One-way ANOVA with multiple comparison testing (Tukey) was performed. Multiplicity adjusted p-values with 95% confidence interval was applied. Adjusted p-values comparing vehicle control without vs. with platelets and compound treatments vs. vehicle control in the presence of platelets are presented in the figure. The stars indicate significant p-values, whereas ns indicates not significant. (**C**,**D**) Invasion assay with SNU-475 cells as described in (**A**) and (**B**). 2.5 µM resminostat and 5 µM sorafenib were used in mono- and combination treatments.

To determine the drug effect of resminostat and sorafenib on platelet-induced cellular invasion, transwell invasion assays were performed for PLC/PRF/5 (Fig. 4A,B) and SNU-475 cells (Fig. 4C,D) with and without platelets and in the presence of resminostat and sorafenib as mono-treatments or in combination. To correct for possible drug toxicity effects, growth controls were performed in parallel to the transwell invasion assays. For quantification of the transwell invasion assay, the OD values of the growth controls were used for normalization. In the DMSO control, platelets significantly induced the invasive capacity of PLC/PRF/5 (p = 0.0003) and SNU-475 cells (p < 0.0001) approximately seven- and four-fold, respectively. Importantly, the invasion assay revealed a significant reduction (PLC/PRF/5 DMSO vs. combination p = 0.0015; SNU-475 DMSO vs. combination p = 0.0041) in platelet-induced cell invasion when both drugs were used in combination, but not when each drug was used as mono-treatment (Fig. 4B,D). This indicates that the drug combination, but not the mono-treatments, effectively counteracts the platelet-induced invasion of HCC cells.

### The resminostat/sorafenib drug combination de-regulates EMT-gene expression and impairs MEK/ERK-signaling

Epithelial-mesenchymal plasticity (EMP), the dynamic process of epithelial-to-mesenchymal (EMT) transition and its reversion, mesenchymal-to-epithelial transition (MET), has been implicated in cancer stem cell generation, therapeutic resistance, as well as effective cancer metastasis, e.g. via contributing to the invasive potential of cancer cells. Moreover, accumulating evidence suggest that cancer cell populations with partial EMT, epithelial features and/or a hybrid epithelial-mesenchymal phenotype retain metastatic capabilities. Ongoing research efforts are aiming to identify cell populations with invasive capacities in cancer^36^.

**Figure 5:**
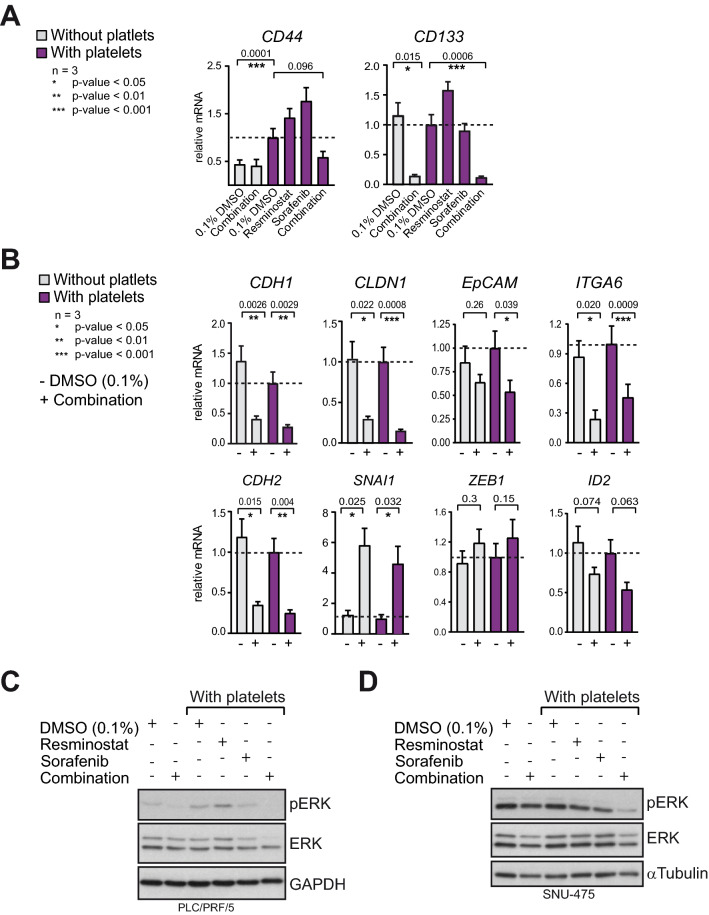
The combination of resminostat and sorafenib de-regulates the expression of EMT genes and decreases pERK level. (**A**) Relative mRNA expression of mesenchymal and cancer stem cell marker *CD44* and *CD133*. PLT/PRF/5 cells were treated with or without platelets and with drug(s) as indicated. Data from three biological replicates are presented as data means with SD. Paired two-tailed t-test was performed. (**B**) Relative mRNA expression of EMT genes *CDH2* (*N-Cadherin*), *SNAI1*, *ZEB1*, *ID2, CDH1* (*E-Cadherin*), *CLDN1, EpCAM* and *ITGA6*. Data from three biological replicates are presented as data means with SD. Paired two-tailed t-test was performed. (**C**) Western blots for total level of the MEK signaling kinase ERK and its activated form pERK (phosphorylated ERK). GAPDH was used as loading control. PLC/PRF/5 cells were cultured with or without platelets and with drugs as indicated. Grouped blots were composed from cropped parts of individual blots for each antibody. Original blots with exposures and indicated cropped areas can be found in the supplementary information. (**D**) Same as in (**C**) but with SNU-475 cells. Staining for α-Tubulin was used as loading control.

In HCC, a cell population expressing the mesenchymal and stem cell-associated marker *CD44* together with the stem cell marker *CD133* has been described as cancer progenitor cells with invasive capacity^37,38^. We observed that co-culture of PLC/PRF/5 cells with platelets induced an increase of the mRNA level of *CD44.* In comparison, *CD133* mRNA level was not increassed by platelets. Importantly, only the drug combination but not the mono-treatments reduced *CD44* and *CD133* mRNA levels (Fig. 5A). Next, the impact on the expression of several EMT genes was explored. The resminostat/sorafenib drug combination considerably de-regulated the expression of epithelial and mesenchymal genes. A directed regulation towards either the epithelial (*CDH1, CLDN1, EpCAM, ITGA6*) or the mesenchymal (*CDH2, SNAI1, ZEB1*) gene expression pattern was not observed (Fig. 5B). Among those genes, *CLDN1, ITGA6 * and * ID*2 have been reported to promote EMT, cell migration and proliferation in liver cancer cells^39–41^, and the resminostat/sorafenib drug combination reduced the mRNA level of these genes (significantly for *CLDN1* p = 0.0008, *ITGA6* p = 0.0009). Moreover, the mitogen-activated Ras/Raf/MEK/extracellular signal‑regulated kinase (ERK) signaling pathway (MEK/ERK) is a central pathway driving tumor growth, cell invasion, migration and EMT^39^. Its aberrant activation is important for the occurrence and development of HCC^42^. Here, it was observed that the drug combination treatment reduced the level of phosphorylated ERK (pERK) level in PLC/PRF/5 and SNU-475 cells, whereas the mono-treatments reduced pERK less effectively. The pERK inihibtion by the drug combination was more pronounced in the presence of platelets, particularily in SNU-475 cells (Fig. 5C,D). In contrast, growth factors which were either released by platelets and detectable in the supernatant, or secreted by HCC cells after stimulation with platelets (Fig. S4A), were not consistantly affected by resminosat, sorafenib or the drug combination (Fig. S4B). Taken together, the resminostat/sorafenib drug combination disrupts the expression of EMT genes, reduces pERK-signalling in the presence of platelets, and may reduce the population of invasive CD44/CD133 positive cells.

### Resminostat in combination with the MKis sorafenib or regorafenib reversed platelet-induced cellular invasion of HCC cells

Other kinase inhibitors, aside from sorafenib, have been clinically developed for the treatment of various cancers and some of them have been recently approved in HCC^2,5^. The multikinase inhibitor regorafenib, approved for second-line treatment of HCC, is structurally similar to sorafenib, but has a broader activity than sorafenib and differs slightly in its inhibitory profile^27^. In addition to inhibiting RAF kinases, it has a greater potency against VEGFR, a receptor tyrosine kinase involved in pathological angiogenesis, and also targets several other kinases, among them TIE2, FGFR1 and RET^43^. The kinase inhibitor lenvatinib, recently approved as first-line treatment in several regions including Europe and the United States, acts more selectively via the inhibition of VEGFR^44^. Moreover, the kinase inhibitor crizotinib was specifically designed to inhibit ALK and c-MET (hepatocyte growth factor receptor, HGFR) and is currently being clinically investigated in several cancer indications^45^.

**Figure 6:**
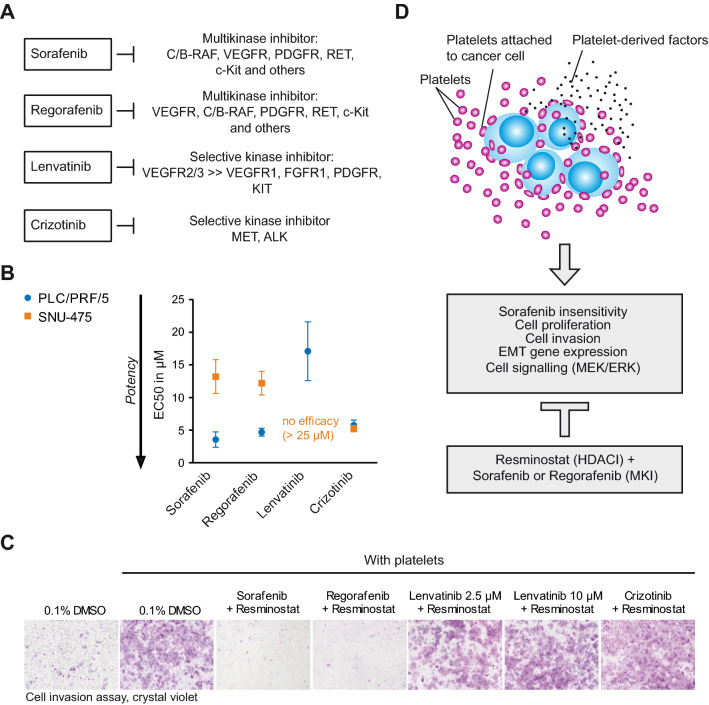
Resminostat in combination with the MKi sorafenib or regorafenib reversed platelet-induced cellular invasion of HCC cells. (**A**) Schematic presentation of the kinase inhibitor profile of sorafenib, regorafenib, lenvatinib and crizotinib. (**B**) EC50 values for the anti-proliferative activities of kinase inhibitors sorafenib, regorafenib, crizotinib and lenvatinib in PLC/PRF/5 and SNU-475 cells. The data are presented as mean ± SD (n = 3). (**C**) Invasion assay of PLC/PRF/5 with platelets. Resminostat in combination with several kinase inhibitors; sorafenib, regorafenib, lenvatinib and crizotinib. A representative experiment is shown. (**D**) Model: Inhibition of platelet-mediated cancer-promoting effects by the drug combination of resminostat and the MKi sorafenib or regorafenib.

Analysis of the efficacies of sorafenib and regorafenib in HCC cells using proliferation assays in epithelial PLC/PRF/5 and mesenchymal SNU-457 revealed similar EC50 values for sorafenib and regorafenib, with higher EC50 values SNU-475 compared to PLC/PRF/5 cells for both kinase inhibitors (Fig. 6A,B). In comparison, lenvatinib was less effective in the PLC/PRF/5 and SNU-475 cell line. With ~ 17 µM, the EC50 of lenvatinib was 4- to five-fold higher than the EC50 for sorafenib and regorafenib in PLC/PRF/5 cells. In SNU-475 cells, an EC50 value for lenvatinib could not be determined due to a lack of efficacy. In contrast to sorafenib and regorafenib, crizotinib had EC50 values (~ 5 µM) comparable in PLC/PRF/5 cells but did not show reduced activity in mesenchymal SNU-457 cells and had similar EC50 values ~ 5 µM in both cell lines (Fig. 6B).

We then compared combinations of resminostat with the aforementioned kinase inhibitors in cell invasion assays (Fig. 6C). The regorafenib/resminostat combination, like the sorafenib/resminostat combination, blocked platelet-induced cellular invasion. In contrast, this was not observed for the combination with lenvatinib or crizotinib, indicating that the inhibition of a certain kinase, or several of them, is required for reducing platelet-induced cell invasion in combination with resminostat. Taken together, these experiments allow the conclusion that the drug combination of resminostat with certain MKis, such as sorafenib or regorafenib, counteracts the platelet-mediated drug resistance and platelet-induced cell invasion of HCC cells (Fig. 6D).

## Discussion

The treatment of advanced, unresectable HCC is limited by a lack of therapeutic options and suitable biomarkers for patient stratification. Our preclinical findings provide a scientific rationale for a platelet count-dependent efficacy of the resminostat/sorafenib drug combination in HCC and highlight blood platelet count as a potential treatment predictive biomarker for MKi/HDACi therapy. Moreover, the data suggest sorafenib or similar MKis as promising combination partners for HDACi such as resminostat in platelet-driven cancers such as HCC.

HDACi have emerged as drug candidates for cancer treatment since deregulated HDAC activities have been described in several cancers^17,46^. As part of the chromatin modifying machinery, HDACs and their enzymatic counterpart, histone acetyltransferases (HATs), regulate the acetylation status of histones and thus contribute to the epigenetic control of gene expression in normal and cancer cells, for instance via EMT. Moreover, HDACs deacetylate a multitude of cytosolic and chromatin-associated proteins, including proteins with roles in cancer as well as cytoskeleton components. The anti-cancer activities of HDACi have been attributed but not limited to the induction of cell cycle arrest, DNA damage, apoptosis and/or the alteration of the cancer cell gene expression signature^17,46,47^. In accordance with its in vitro inhibitory profile, resminostat causes hyperacetylation of a histone and a non-histone substrate in HCC cells. Moreover, complementing previous reports^22–24^, we demonstrated that resminostat has anti-proliferative activities in HCC cells and in several other cancer indications. In an orthotopic xenograft mouse model for liver cancer, resminostat and sorafenib were more effective in reducing tumor growth in vivo when administered in combination. Similar observations have been made for other HDACi, such as quisinostat, panabinostat and MPT0E028, in HCC mouse models^48–50^.

However, recent clinical trial data in HCC indicate that platelet count is linked to the outcome of treatment with the resminostat/sorafenib combination or with sorafenib alone. To date, the phase I/II clinical trial NCT02400788 is the only completed and reported clinical trial addressing the efficacy of an HDACi in combination with sorafenib in a first-line setting. Evaluation of the trial data revealed that the drug combination resulted in longer TPP and OS compared to the sorafenib mono-arm in patients with baseline platelet counts ≥ 150 × 10^3^/mm^3^, but not below that threshold. Moreover, sorafenib mono-treatment showed a reversed trend^21^. In line with these findings, retrospective analysis of the Asian Pacific (AP) and SHARP trial revealed that patients with platelet counts > 150 × 10^3^ PLTs/mm^3^ had an impaired therapeutic response to sorafenib compared to patients with lower platelet counts^51^. Such a platelet count-dependent outcome might be explained by either pro-tumoral features of platelets that cannot be overcome by sorafenib alone, or platelet-mediated drug insensitivity towards sorafenib, or by both these mechanisms.

In vitro findings in this manuscript and other studies demonstrated that platelet factors abolish the growth-inhibitory and pro-apoptotic action of sorafenib and regorafenib^15^. Sorafenib and regorafenib inhibit at least 14 tyrosine and serine/threonine kinases, including Raf-kinases and several cell surface kinase receptors, resulting in the inhibition of the growth and invasion promoting MEK/ERK signalling and other pathways^52–54^. The release of growth factors, such as PDGF, VEGF, EGFs, and TGFβ, which activate their cognate growth factor receptors on surrounding cancer or tissue cells, is an established platelet-mediated signalling mechanism^7,8,12^. Indeed, the Raf-MEK-ERK mitogen-activated protein kinase cascade was found to be activated by platelet lysate in vitro, outlining the possbility to cause the sorafenib insensitivity^15,55^. Importantly, we demonstrate here that the sensitivity to resminostat was not diminished by platelet lysate, indicating an alternative anti-proliferative mechanism of resminostat compared to sorafenib. However, considering that a beneficial effect by resminostat mono-treatment compared to the drug combination was not observed in previous clinical trials, effects other than growth inhibition likely account for the platelet-dependent combinatory benefit^20,21^.

A key cancer-promoting effect of platelets is to facilitate cell invasion, EMT and metastasis^12,13^. We observed that treatment of cancer cells with platelets, but not with platelet lysate, induced cell invasion in HCC cells, implicating the involvement of the membrane fragments of platelets. Similarily, a direct interaction of platelets with breast cancer cells induced an epithelial-to-mesenchymal (EMT)-like invasive phenotype and primed cancer cells for metastasis, while activation of TGFβ- or NFκB-signalling alone was not sufficient to produce this effect^35^. In the present work, alterations in the levels of the growth factors PDGF or TGFβ or VEGF upon treatment with neither resminostat nor sorafenib were detected, pointing towards a mechanism not related to an alteration of growth factor release or secretion. An association between cancer cells and platelets has been described in undifferentiated liver tumor tissue, suggesting a direct interaction might be physiologically relevant in the tumor^56^. However, the in vivo relevance of platelets for EMT and cell invasion is not entirely understood and remains controversial^30,57,58^. In future studies, it will be interesting to investigate whether blood platelets are directly causative of modulating EMP, cell invasion, and metastasis in vivo in HCC, and whether this can be reversed by the resminostat/sorafenib drug combination.

We uncovered that the combination of resminostat and sorafenib, but not the respective mono-treatments, effectively counteracted platelet-induced cellular invasiveness of HCC cells. However, while for Sorafenib a negative effect on cell invasion or migration has been reported^59,60^, no significant effect of sorafenib on platelet-induced cell invasion was detected in our assay. Providing mechanistic insight in the interruption of platelet-induced cell invasion by the drug combination, we observed that expression of *CD44*, a stemness and mesenchymal marker, was induced by platelet treatment of HCC cells. Interestingly, recent evidence suggest that CD44^+^/CD133^+^ HCC cells define a cell population particularly responsible for hematogenous metastasis in liver cancer^37,38^. Moreover, CD44 expression in the background of a mesenchymal-like HCC cells phenotype was refractory to sorafenib-induced cell death^33^. Importantly, only the drug combination but not the mono-treatments reduced the platelet-induced increase of *CD44* mRNA level. Moreover, the drug combination considerably reduced the expression of the stem cell marker *CD133,* which was previously found to be expressed in epithelial-like HCC cells^33^. Thus, our findings allow the assumption that the resminostat/sorafenib drug combination might reduce the invasive CD44^+^/CD133^+^ tumor cell population in HCC.

EMP is a dynamically regulated process and HDAC proteins contribute to the execution of the underlying EMT/MET gene expression program^36,47^. Moreover, biomarker analysis of HCC tumors revealed an association between mesenchymal marker gene expression and shorter disease-free survival. The mesenchymal phenotype and an enhanced metastatic potential have been found in HCC cell lines insensitive to Sorafenib^29,33^. In agreement with resminsotat-mediated deregulation of EMT gene expression in HLE und HLF cells^23^, we observed that combination treatment with resminostat and sorafenib deregulated the expression of several epithelial and mesenchymal genes, even in the presence of platelets. Taken together, these findings suggest that resminostat disrupts the regulation of the EMT program and affects EMP.

The deregulation of EMT genes by resminostat may be mediated via the inhibition of class I HDACs. HDAC1 and 2 are known to be important transcriptional regulators that act by removing acetyl-lysine residues from histone tails (e.g. H3K27ac and H3K9ac), thus antagonizing the activity of histone acetyltransferases (p300/CBP)^26,47,61^. In EMT, it has been reported that the mesenchymal transcription factors Snail and ZEB recruit HDAC repressor complexes to chromatin to silence E-Caderin^62^. In addition to the role in transcriptional repression, several lines of evidence have revealed that HDAC1/2 complexes also play a role in active transcription, e.g. through cyclical HDAC/HAT activities on the chromatin^63^. Moreover, sorafenib and resminostat may exert their effects via modulating the activity of transcription factors; sorafenib by blocking the MEK/ERK-mediated phosphorylation of transcription factors and resminostat by promoting hyperacetylation of transcription factors, thus altering their binding to DNA, stability or association with co-activating proteins^19,26,47^.

Furthermore, we provide evidence that the combined drug effect involves the MEK/ERK signalling pathway, a mitogen signalling pathway described to promote cell growth and cell invasion^64^. Interestingly, linking HDAC6 function and MEK/ERK signalling, a previous report proposed that the MEK/ERK signalling pathway promotes cell migration in part through phosphorylation of HDAC6, thereby enhancing HDAC6 deacetylase activity towards α-TUBULIN^65,66^. Considering the multiple targets of resminostat and sorafenib, respectively, both drugs may collaborate on multiple levels, resulting in the disruption of platelet-initiated signalling pathways and gene expression programs.

Platelet count is an easy to assess standard blood value and our data underline its potential as a predictive marker for the treatment with resminostat and sorafenib in HCC. Interestingly, in HCC, the pre-operative platelet count has already been recognized to have prognostic value for the survival of HCC patients^10,67^. The platelet count covers ranges from pathologically low in thrombocytopenia to normal platelet counts of  ≥ 150 × 10^3^ PLTs/mm^3^, up to abnormally high levels in thrombocytosis > 400 × 10^3^ PLTs/mm^3^^8,68^. Although thrombocytopenia has been reported to coincide with lower tumor sizes, it is a severe hematological complication of chronic liver disease and is associated with a higher risk of death^68,69^. In addition, HCC patients with thrombocytopenia (< 60 × 10^3^ PLTs/mm^3^) are considered unsuitable for treatment with sorafenib due to the risk of bleeding^68,70^. In contrast, patients with platelet counts ≥ 150 × 10^3^/mm^3^ and with thrombocytosis are being diagnosed with larger tumors and have a higher risk of extrahepatic disease progression^8,9^. Importantly, these patients might be more suitable for the resminostat plus sorafenib combination.

Taken together, we demonstrate that the drug combination of the broad-spectrum HDACi resminostat with the MKi sorafenib counteracts platelet-mediated pro-tumoral effects in HCC cells. Considering both our in vitro findings and the evaluation of clinical data, baseline platelet count might be a valuable predictive biomarker for the use of the drug combination resminostat with sorafenib in HCC. Importantly, a prospective randomized phase III study is needed to verify the baseline platelet count as such predictive marker for the drug combination. Such a clinical trial will possibly generate transferable results for other platelet-driven diseases.

## Materials and methods

### Cell lines and cell culture

The cell lines HepG2 (ACC180, RRID:CVCL_0027; DMSZ, Braunschweig Germany), SNU-475 (CRL-2236, Lot. 58483227, RRID:CVCL_0497; ATCC, Manassas, Virginia, US), SNU-387 (CRL-2237, Lot. 58483192, RRID:CVCL_0250; ATCC, Virginia, US), PLC/PRF/5 (300315, Lot. 812, RRID:CVCL_0485; CLS Cell Lines Service GmbH, Eppelheim, Germany) and HuH-7 (JCRB0403, Lot. 2272008, RRID:CVCL_0336; JCRB Cell Bank, Japan) were propagated according to the distributor’s guidelines. The authenticity of the cell lines was confirmed by short tandem repeat (STR) analysis (DSMZ, Braunschweig, Germany). Mycoplasma contamination was ruled out by PCR testing. For assays with platelet lysate (PL), FBS was substituted with 10% PLTMax Human Platelet Lysate (Cat. Nr. SCM141, Lot Number VP1602259; Millipore, Burlington, Massachusetts, US). The PL was supplemented with 20 U/ml Heparin (Heparin sodium salt from porcine intestinal mucosa, H4784-250MG; Sigma-Aldrich, Taufkirchen, Germany) to prevent coagulation.

### Drugs (compounds)

Resminostat (Product Code BYK408740, CAS Nr: 934004-03-2; supplied by 4SC AG, Planegg, Germany); Sorafenib (Nexavar), (Product Code S-8502, CAS No 4750207-59-1; LC Laboratories, Woburn, Massachusetts, US); Regorafenib (Stivarga), (Product Code GP6787, Cas No 755037-03-7; ChemPur GmbH, Karlsruhe, Germany); Lenvatinib (Lenvima), (Product Code E7080, Cas No 417716-92-8; Activate Scientific GmbH, Prien am Chiemsee, Germany); Crizotinib (Xalkori), (Product Code S1068, Cas No 877399-52-5; Selleck Chemicals Co. Ltd., Munich, Germany). All drugs were dissolved in DMSO.

### Orthotopic xenograft mouse model

The orthotopic xenograft mouse model of Hep3B2.1–7 human hepatic carcinoma was previously established^28^. Tumors were created by injection of 2.5 × 10^6^ Hep3B2.1-7 cells (ATCC, RRID:CVCL_0326; Virginia, Manassas, USA) into the main lobe of the liver of anaesthetized female SCID mice (C.B-17-Igh-1bPrkdc(scid)). Fifteen days post-inoculation, the take-rate of the tumors was 80% and oral treatment with either vehicle control (NMP:PEG300:Saline, 1:9:10 v/v); resminostat (15 mg/kg and 40 mg/kg), sorafenib (15 mg/kg), or the resminostat/sorafenib combination (15/15 mg/kg, 40/15 mg/kg) commenced. Treatments were continued for 21 days. Treatment of animals was ceased when the body weight dropped below 85% of that on entry of the study. Only mice culled at the termination date were used to determine the tumor weight. The experiment was conducted by vivoPharm pty Ltd (Bundoora, Australia) in accordance with the principles outlined in the “Australian Code of Practice for the Care and Use of Animals for Scientific Purposes”, 7th Edition, 2004 (National Health and Medical Research Council, Canberra, Australia). All procedures involving animals were reviewed and approved by the University of Adelaide Animal Ethics Committee. The study was carried out in compliance with the ARRIVE guidelines (http://www.nc3rs.org.uk/page.asp?id=1357).

### RNA sample preparation and realtime PCR

To generate cDNA, total RNA was extracted from cells using the RNeasy Purification Kit (#74106; Qiagen, Hilden, Germany) and the RNase-Free DNase set (#79254; Qiagen, Hilden, Germany). The RNA was then reversed transcribed using the M-MLV Reverse Transcriptase (#28025013; Promega, Fitchburg, Wisconsin, US). Relative mRNA expression levels were determined by realtime PCR using SYBR Green I detection chemistry (#A6002; Promega, Wisconsin, Fitchburg, US). Data were analyzed using the ΔΔCT method, with housekeeping gene (*GAPDH*) as the normalizer^71^.

### Proliferation assay and EC50 determination

Prior to the assay, the optimal seeding density was determined for each cell line to obtain an exponential growth during the experiment (2000 to 6000 cells per 96-well). Drug dilution series with 8 concentrations were prepared for EC50 determinations of single drugs. Three wells for each concentration were seeded (technical replicates). For drug combinations, dilution series of six concentrations for each drug alone or in combination at equal ratio were prepared. After 4 h of cell seeding, the drugs were added to the cells. Each 96-well plate contained a vehicle control (0.1% or 0.2% DMSO), a death control Ro 106-9920 (Tocris Cookson Ltd., Bristol, UK) a growth control (untreated cells compared to vehicle treated cells), and blank wells without cells. The proliferation assay was stopped after 4 days by crystal violet staining of the cells (0.5% in 20% methanol). Following extraction of the crystal violet with 8% acetic acid, the eluates were quantified photometrically at 570 nm and the reference wavelength 690 nm in an absorbance microplate reader (Tecan Sunrise; Tecan, Männedorf, Switzerland). Only readouts from 96-well plates with a Z-factor between 0.5 and 1.0 were used. Blank OD values were subtracted from the mean OD values. Nonlinear curve fitting and EC50 calculation was performed with Excel Solver.

### Isolation of blood platelets

Blood platelets were isolated under sterile conditions from human whole blood. The protocol was adapted from the Abcam protocols (https://www.abcam.com/protocols/isolation-of-human-platelets-from-whole-blood). Platelets from two to three blood donors were combined. The required number of platelets was activated by addition of 0.5 U/ml thrombin (Ref. 10602400001; Roche, Switzerland) for 15 min at 37 °C.

### Cell invasion assay

The protocol was performed with some alterations according to the manufacturer’s protocol and similar as previously described^35^. Permeable transwell invasion chambers with 8 µm pore size (#353097; Corning, New York, US) and 24-well companion plates (#353504; Corning, New York, US) were used. Prior to the assay, the cells were subcultured either in normal growth medium or with platelets for several days. 2 h before the assay was set up, the transwell chambers were coated with 300 µg/mL Matrigel basement membrane matrix (#354234; Corning, New York, US). Then, cell suspensions with or without platelets, drugs, or 0.1% DMSO vehicle control, were transferred into the transwell chambers. The drug concentrations used in the assay were adapted to the drug sensitivities of the cell lines based on their EC50 values. At the 3rd day of incubation, the assay was stopped by crystal violet staining (0.5% in 20% methanol). Microscopic images were taken at two-fold magnification prior to elution of the crystal violet with 8% acetic acid (Olympus IX50 microscope, Camera Olympus SC30; Olympus, Tokyo, Japan). Eluates were photometrically quantified at 570 nm with the reference wavelength 690 nm using an absorbance microplate reader (Tecan Sunrise; Tecan, Männedorf, Switzerland). In parallel to the invasion assay, the same experimental setup was seeded in tissue culture plates without the transwell chambers. This served as a control for toxicity and growth during the assay. The ODs of the transwells were normalized to the ODs from tissue culture plates.

For more Materials and Methods, see supplementary information.

## Supplementary Information

Supplementary Information 1.

Supplementary Information 2.

## Data Availability

The data that were generated during the current study are available from the corresponding author on reasonable request. Data that support the findings are available in the supplementary information.
