# Supplementary material for: Histone deacetylase inhibitor resminostat in combination with sorafenib counteracts platelet-mediated pro-tumoral effects in hepatocellular carcinoma
Source: Sci Rep. 2021 May 5;11:9587. doi: 10.1038/s41598-021-88983-1 (PMC8100298; doi:10.1038/s41598-021-88983-1)
Supplement: Supplementary file 1 — Supplementary Information 1. [file 41598_2021_88983_MOESM1_ESM.pdf]

Supplementary information

Title Page

Title

Histone deacetylase inhibitor resminostat in combination with sorafenib counteracts platelet-mediated pro-tumoral effects in hepatocellular carcinoma.

Authors and affiliations

Gundula Streubel[1*], Sabine Schrepfer[1], Hannah Kallus[1], Ulrike Parnitzke[1], Tanja Wulff[1], Frank Hermann[1], Matthias Borgmann[1], Svetlana Hamm[1]

[1]4SC AG, Fraunhoferstraße 22, 82152 Planegg-Martinsried, Germany

Supplemental Figures and Figure legends

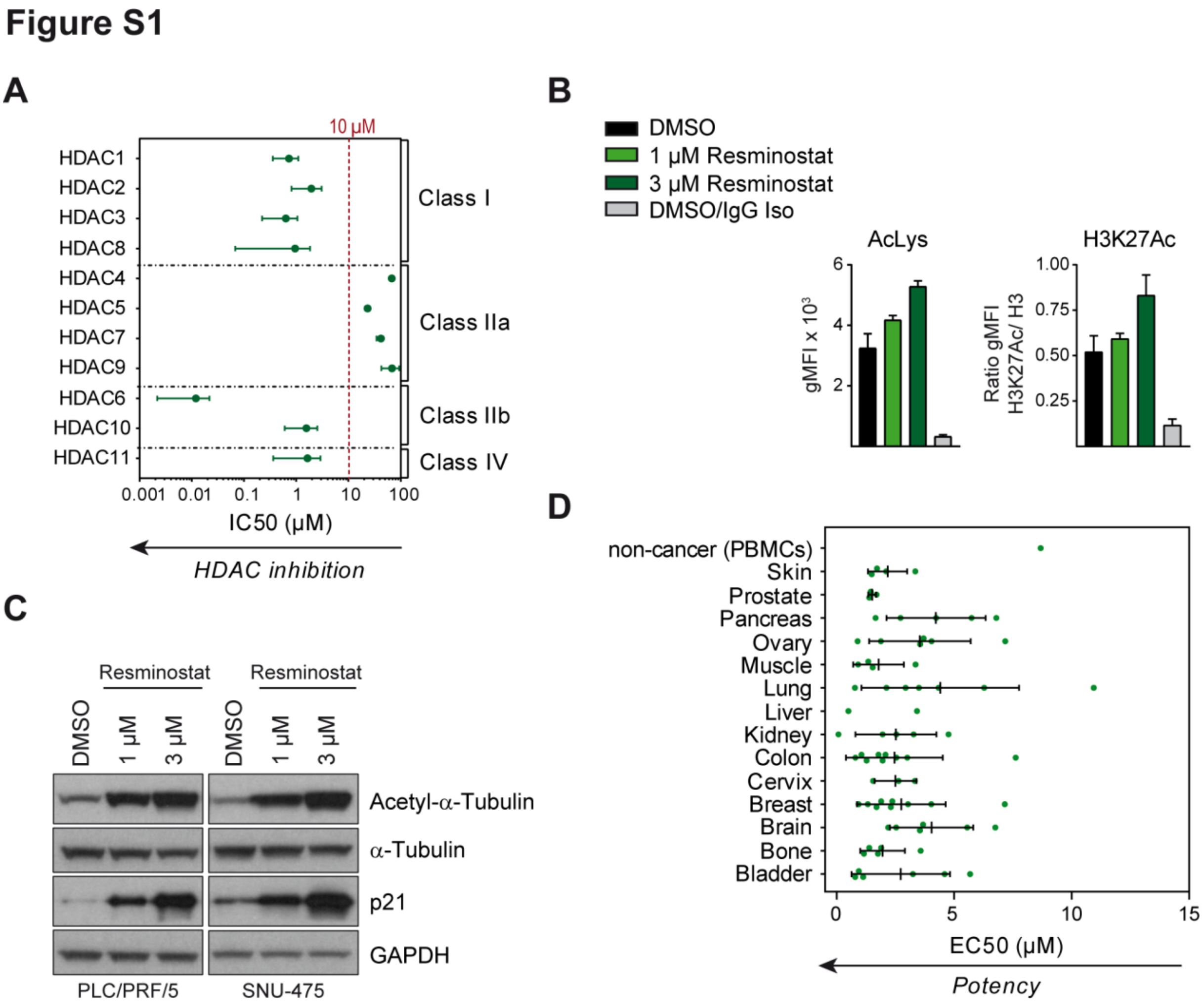

Figure S1. Inhibition of cancer cell growth by the broad-spectrum HDACI resminostat

A. Inhibition of HDAC1 to 11 by resminostat determined by in vitro HDAC activity assays. IC50 data from three independent experiments are shown as mean ± SD.

B. Intracellular flow cytometry using antibodies targeting acetyl lysine (AcLys) and the H3K27 acetylation mark (H3K27Ac). IgG isotype controls were used for each antibody. PLC/PRF/5 HCC cells were treated with vehicle control (0.1 % DMSO), 1 µM or 3 µM resminostat for 24 hours. For AcLys, the geometric mean fluorescence intensities (gMFI) are shown. For H3K27Ac, the ratios of the gMFIs for H3K27Ac relative to Histone H3 are shown. Data of three technical replicates are presented as mean ± SD.

C. Western blots for acetylated α-Tubulin, total α-Tubulin, p21 and GAPDH (loading control). Cells were treated with vehicle control (0.1 % DMSO), 1 µM or 3 µM resminostat for 24 hours. Grouped blots were composed from cropped parts of individual blots for each antibody. Original blots with exposures and indicated cropped areas can be found in the supplementary information.

D. Cell line screen determining the EC50s of resminostat in 74 cell lines representing 14 different cancer indications. In addition, peripheral blood mononuclear cells (PBMCs) from healthy donors were tested. Each dot represents an EC50.

Figure S2

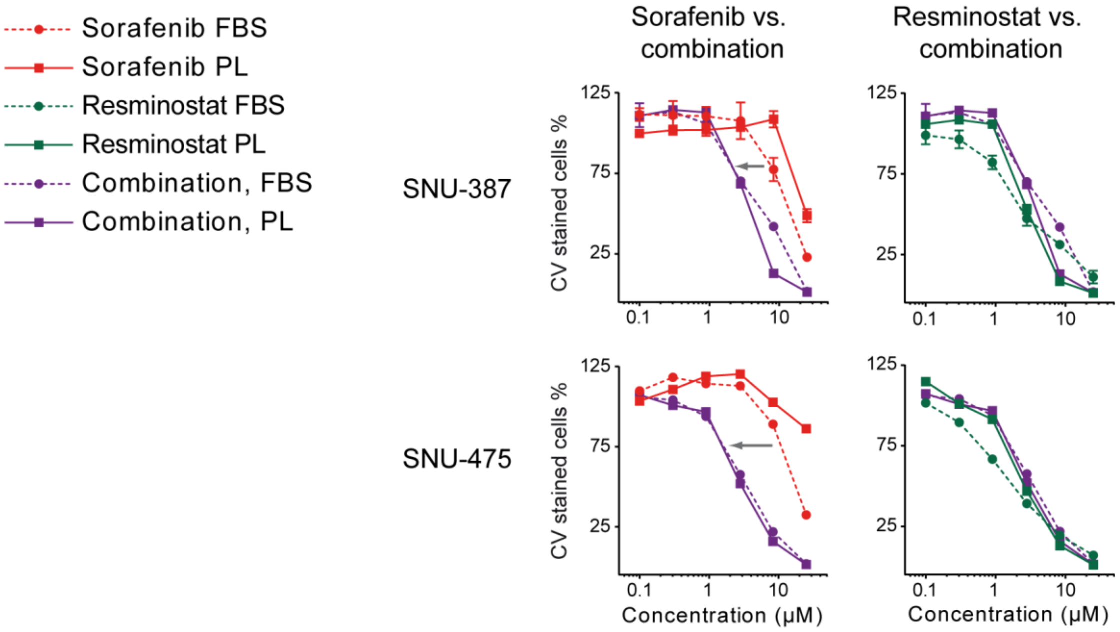

Figure S2. Anti-proliferative activity of resminostat in mesenchymal HCC cells in the presence of platelet lysate

Proliferation assays and dose-response curves in SNU-475 and SNU-387 cells in the presence of fetal bovine serum (FBS) or platelet lysate (PL). Drug concentration ranged from 0.1 to 25 µM. The grey arrow indicates a difference in the potency of the drug combination compared to sorafenib.

Figure S3

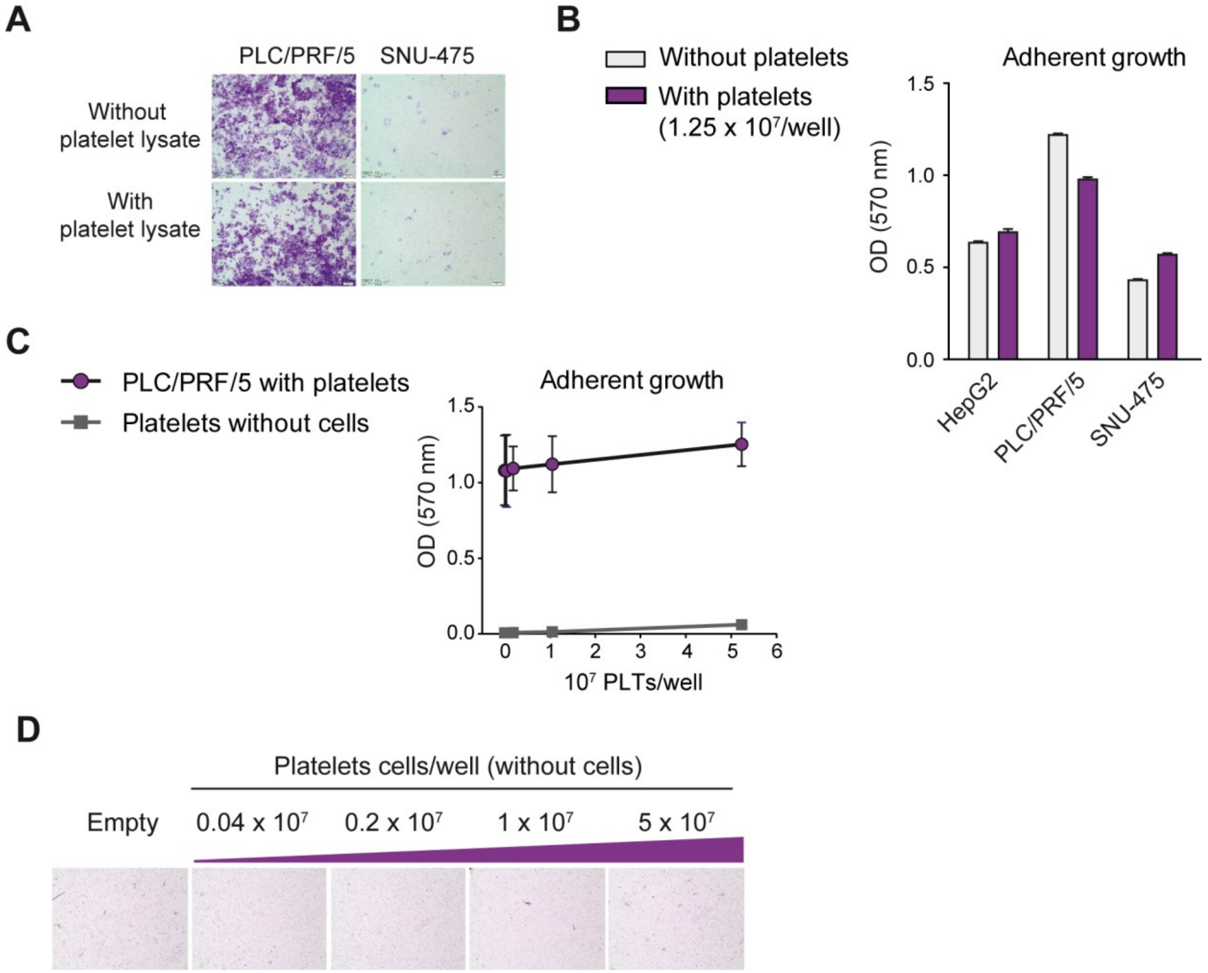

Figure S3. Controls for transwell invasion assay

A. Transwell invasion assay for PLC/PRF/5 and SNU-475 cells cultured either with 10% platelet lysates or with 10% FBS (without PL) for several days prior to seeding of the assay.

B. Adherent growth of HepG2, PLC/PRF/5 and SNU475 cells with and without platelets. The same proportions of cells, platelets, and growth media with chemoattractant as in the cell invasion assays were used. Data from technical replicates are shown as mean with SD.

C. Adherent cell growth with increasing platelet numbers. Cells were cultured in parallel to Figure 3C in cell culture plates. The same proportions of cells, platelets, drugs and growth media with chemoattractant as in the cell invasion assay were used. ODs were determined at day 3 upon cell seeding. Data are shown as mean ± SD (n = 3).

D. Invasion assay with increasing numbers of platelets seeded without cells. Microscopic images of crystal violet stained transwells.

Figure S4

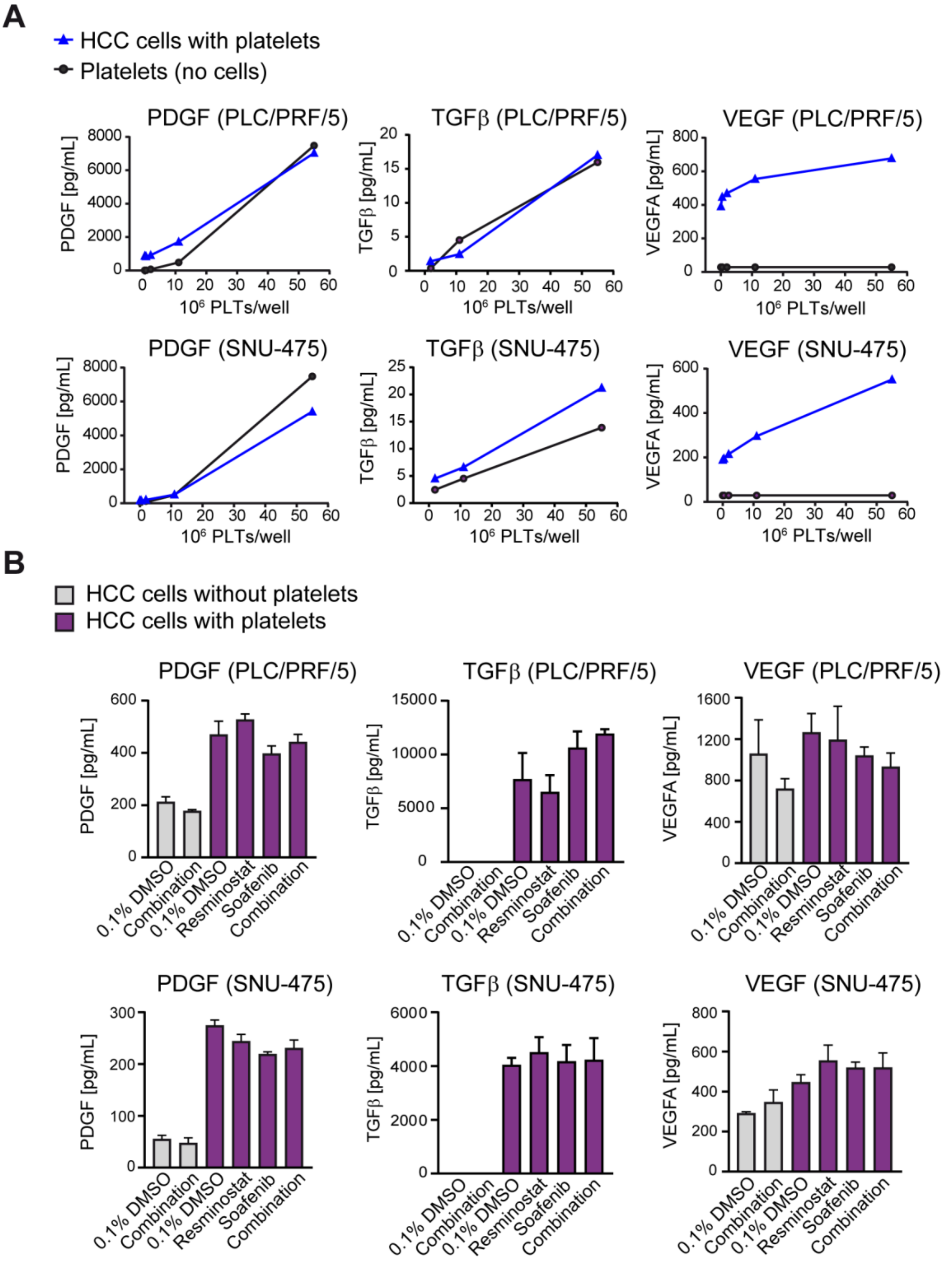

Figure S4. Resminostat and sorafenib did not inhibit growth factor release

A. The growth factors PDGF-AA, TGFβ and VEGF were determined from tissue culture supernatants by ELISA. PLC/PRF/5 and SNU-475 cells were cultured with increasing

amounts of platelets. In parallel, platelets were cultured without cells to determine if the growth factors were platelet derived.

B. ELSIAs for PDGF-AA, TGFβ and VEGF were performed after drug treatment of PC/PRF/5 and SNU-475 cells. Cells were cultured as indicated with or without platelets and treated with resminostat, sorafenib, the drug combination or vehicle control 0.1% DMSO.

Table S1_ EC50 cell line screen

Table summary of the efficacy proliferation screening assay for resminostat shown in Figure S1D. The cancer cell lines, their tissue origins and the respective EC50 values are shown. Each cell line was seeded in duplicates.

Supplementary Materials and Methods

Antibodies

For western blotting, the primary antibodies p21 WAF1/CIP1 rabbit mAb (#2947, clone ID 12D1; CST, Massachusetts, US), TUBA1A (α-Tubulin) mouse mAb (66031-1-Ig, clone ID 1E4C11; Proteintech Group Inc, Illinois, US), Acetyl-TUBA1A (Lys40) rabbit mAb (#5335, clone ID D20G3; CST, Massachusetts, US), phospho-p44/42 MAPK (Erk1/2) rabbit pAb (#9101, Cell Signalling) and p44/42 MAPK (Erk1/2) rabbit pAb (#9102, Cell Signalling) and GAPDH rabbit mAb (ab128915, clone ID EPR6256; Abcam, UK) were used. HRP-linked goat anti-rabbit IgG (111-035-003; Jackson Laboratory, Maine, US) and HRP-linked goat anti-mouse IgG (115-035-003; Jackson Laboratory, Maine, US) were used as the secondary antibodies. For flow cytometry analysis, an acetyl-Lysine mouse mAb (# 9681S, clone ID Ac-K-103; CST, Massachusetts, US) was used together with a goat anti-mouse IgG2a cross-adsorbed secondary antibody linked with Alexa Fluor 488 (# A21131; Life technologies, California, US); an Histone H3K27ac rabbit pAb (# 39133; Active motif, California, US) and an Histone H3 antibody rabbit pAb (ab1791; Abcam, UK) were used with goat anti-rabbit IgG linked with Alexa Fluor 488 (# 150077; Abcam, UK). The mouse IgG2a isotype control (#M5409, clone ID UPC-10; Sigma-Aldrich, Missouri, US) and normal rabbit IgG (#2729S, CST, Massachusetts, US) were used as the control antibodies.

**Antibody**	**RRID**	**Citation**
p21 WAF1/CIP1, rabbit mAb 12D1, #2947	AB_823586	Deng, L. et al. (2019) “Stabilizing heterochromatin by DGCR8 alleviates senescence and osteoarthritis,” Nature Communications, 10(1), p. 3329. doi: 10.1038/s41467-019-10831-8.
TUBA1A mouse mAb 1E4C11, 66031-1-Ig	AB_11042766	Zhu, C. et al. (2015) “SUMOylation at K707 of DGCR8 controls direct function of primary microRNA.,” Nucleic acids research. England, 43(16), pp. 7945–7960. doi: 10.1093/nar/gkv741.
Acetyl-TUBA1A (Lys40) rabbit mAb D20G3, #5335	AB_10544694	Mo, Z. et al. (2018) “Aberrant GlyRS-HDAC6 interaction linked to axonal transport deficits in Charcot-Marie-Tooth neuropathy.,” Nature communications. England, 9(1), p. 1007. doi: 10.1038/s41467-018-03461-z.
GAPDH rabbit mAb	AB_11143050	Gao, Q. et al. (2019) “Heterotypic CAF-tumour

Antibody	RRID	Citation
EPR6256, ab128915		spheroids promote early peritoneal metastasis of ovarian cancer.," The Journal of experimental medicine. United States, 216(3), pp. 688–703. doi: 10.1084/jem.20180765.
phospho-p44/42 MAPK, rabbit pAb, #9101	AB_331646	Xu Y, Wang YQ, Wang AT, et al. Effect of CD44 on differentiation of human amniotic mesenchymal stem cells into chondrocytes via Smad and ERK signalling pathways. Mol Med Rep. 2020;21(6):2357-2366. doi:10.3892/mmr.2020.11044
p44/42 MAPK, rabbit pAb, #9102	AB_330744	Ogasawara R, Sato K, Matsutani K, Nakazato K, Fujita S. The order of concurrent endurance and resistance exercise modifies mTOR signalling and protein synthesis in rat skeletal muscle. Am J Physiol Endocrinol Metab. 2014;306(10):E1155-E1162. doi:10.1152/ajpendo.00647.2013
HRP-linked goat anti-rabbit IgG, 111-035-003	AB_2313567	Chuang, S.-M. et al. (2019) "Epigenetic regulation of COX2 expression by DNA hypomethylation via NFkappaB activation in ketamine induced ulcerative cystitis.," International journal of molecular medicine. Greece, 44(3), pp. 797–812. doi: 10.3892/ijmm.2019.4252.
HRP-linked goat anti-mouse IgG, 115-035-003	AB_10015289	Cloud, V. et al. (2019) "Ataxin-7 and Non-stop coordinate SCAR protein levels, subcellular localization, and actin cytoskeleton organization.," eLife. England, 8. doi: 10.7554/eLife.49677.
acetyl-Lysine mouse mAb, clone ID Ac-K-103, #9681S	AB_331799	Da Costa, E. M. et al. (2019) "Heart failure drug proscillaridin A targets MYC overexpressing leukaemia through global loss of lysine acetylation.," Journal of experimental & clinical cancer research: CR. England, 38(1), p. 251. doi: 10.1186/s13046-019-1242-8.
Histone H3K27ac rabbit pAb, # 39133	AB_2722569	Brach, D. et al. (2017) "EZH2 Inhibition by Tazemetostat Results in Altered Dependency on B-cell Activation Signalling in DLBCL.," Molecular cancer therapeutics. United States, 16(11), pp. 2586–2597. doi: 10.1158/1535-7163.MCT-16-0840.
Histone H3 antibody rabbit pAb, ab1791	AB_302613	Sokolova, M. et al. (2017) "Genome-wide screen of cell-cycle regulators in normal and tumour cells identifies a differential response to nucleosome depletion.," Cell cycle (Georgetown, Tex.). United States, 16(2), pp. 189–199. doi: 10.1080/15384101.2016.1261765.
goat anti-mouse IgG2a linked with Alexa Fluor 488, # A21131	AB_2535771	Krutzik, P. O. and Nolan, G. P. (2003) "Intracellular phospho-protein staining techniques for flow cytometry: monitoring single cell signalling events.," Cytometry. Part A: the journal of the International Society for Analytical Cytology. United States, 55(2), pp. 61–70. doi: 10.1002/cyto.a.10072.
goat anti-rabbit IgG linked with Alexa Fluor 488, # 150077	AB_2630356	Pankov, D. et al. (2017) "In vivo immuno-targeting of an extracellular epitope of membrane bound preferentially expressed antigen in melanoma

Antibody	RRID	Citation
		(PRAME).," Oncotarget. United States, 8(39), pp. 65917–65931. doi: 10.18632/oncotarget.19579.
mouse IgG2a isotype control, #M5409	AB_1163691	Brittain, G. C. and Gulnik, S. (2017) "A rapid method for quantifying cytoplasmic versus nuclear localization in endogenous peripheral blood leukocytes by conventional flow cytometry.," Cytometry. Part A: the journal of the International Society for Analytical Cytology. United States, 91(4), pp. 351–363. doi: 10.1002/cyto.a.23103.
normal rabbit IgG, #2729S	AB_1031062	Weissmiller, A. M. et al. (2019) "Inhibition of MYC by the SMARCB1 tumour suppressor.," Nature communications. England, 10(1), p. 2014. doi: 10.1038/s41467-019-10022-5.

Oligo sequences for mRNA

Gene	Forward	Reverse
CD44	AGAAGGTGTGGGCAGAAGAA	AAATGCACCATTTCCTGAGA
CDH1	CAACCCTGCAATCACTTTTTGG	CACAATTATCAGCACCCACACA
EPCAM	TGTCATTTGCTCAAAGCTGG	AAAGCCCATCATTGTTCTGG
GAPDH	GTCAGTGGTGGACCTGACCT	TGCTGTAGCCAAATTCGTTG
RPS18	GAGGATGAGGTGGAACGTGT	TCTTCAGTCGCTCCAGGTCT
SNAI2	TCGGACCCACACATTACCTTG	AAAAAGGCTTCTCCCCCGT
SNAI1	GTCTGACCGATGTGTCTCCC	TGTAAACATCTTCCTCCCAGGC
CDH2	GGTGGAGGAGAAGAAGACCAG	GGCATCAGGCTCCACAGT
CLDN1	TTGACTCCTTGCTGAATCTGAG	GGCCACAAAGATTGCTATCAC
PROM1/ CD133	GCATTGGCATCTTCTATGGTT	CGCCTTGTCCTTGGTAGTGT
ZEB1	CGCAGTCTGGGTGTAATCGT	AGCCAGAATGGGAAAAGCGT
ID2	TGTCAAATGACAGCAAAGCAC	GTTGTTGTTGTGCAAAGAATAAAAG
ITGA6	GGAACTTGTGTCCCTAATGTGTT	CACAGGTCTAAAAACACTGTCAAAG

Western Blot

Protein lysates were generated by lysing cells in IPH buffer (50 mM Tris pH 8.0, 150 mM NaCl, 5 mM EDTA, 0.5% NP40) supplemented with protease/phosphatase inhibitor cocktail (100×, 78440; Thermo Scientific, Massachusetts, US). Following incubation for 1 h at 4°C, lysates were spun for 20 min at 13 000 rpm, 4°C. The protein concentration of the supernatants was determined using the Pierce BCA Protein Assay Kit (#23225; Thermo Fisher Scientific, Massachusetts, US). Equal amounts of protein lysates and a pre-stained protein size marker (Precision Plus Protein Dual Colour Standard; #161-0374; Bio-Rad Laboratories, California, US) were separated by SDS-PAGE (Criterion XT Precast Gel 12% Bis-Tri, #3450118; XT MOPS Running buffer, #161-0788; Bio-Rad Laboratories, California, US) and transferred to PVDF membranes. The membranes were probed using the relevant primary and secondary antibodies according to the manufacturer's instructions. Relative protein levels were determined by chemiluminescence using the Pierce ECL Western blotting substrate (#1859698, #46430; Thermo Fisher Scientific, Massachusetts, US) and exposure of the blots to X-ray films. For the generation of figures, the X-ray films were scanned at least with 300 dpi. Images were processed with GIMP 2.10.12 to generate greyscale images and to adjust brightness and contrast across entire images. No other image manipulations were used. For grouped figures, cropped parts of individual blots for each antibody were composed. Original blots with exposures can be found in the supplement information.

Flow cytometry

One day after seeding, cells were treated with compound for 24 h and then harvested for intracellular FACS staining. For Ethanol fixation, the cells were resuspended in 250 µL PBS, then mixed with 750 µL 100% Ethanol (chilled at -20°C) and incubated at least 30 min on ice. After washing cell with in 500 µl 1%BSA/PBS, cells were made permeable by resuspending

the cell pellet in 500 µl 0.25% Triton-X-100/PBS followed by 7 min incubation at RT. After washing with 400 µL 5%BSA/PBS, cells were stained with primary antibody in 50 µL 1%BSA/PBS for 1 h at RT in the dark. Then, cells were washed in PBS, incubated with secondary fluorescence labelled antibodies for 45 min in the dark at RT, washed twice in PBS to remove the residual antibody and then resuspended in 120 µL 3%FBS/PBS. The cells were measured using the Miltenyi MACSQuant Analyser 10 (Miltenyi) and analysed with the FlowJo Software.

Isolation of blood platelets

The blood was drawn from healthy donors in Becton Dickenson Vacutainers with ACD (acid-citrate-dextrose). To prevent activation of the platelets during the procedure, strong mechanical force was avoided and platelet inhibitor Prostaglandin E1 (PGE1), (Ref. P5515; Sigma-Aldrich, Missouri, US) was added. After spinning at 200 g without applied break for 20 min, the platelet-rich plasma was isolated and supplemented with PGE1 (1 µM final concentration). Next, to remove contamination with red and white blood cells, the plasma was spun at 100 × g for 20 min at RT and the supernatant was transferred into a new tube. The platelet-containing supernatant was spun at 800 × g for 20 min at RT, the supernatant was removed, and the platelet pellet was gently rinsed once with platelet wash buffer (10 mM sodium citrate, 150 mM NaCl, 1 mM EDTA, 1% (w/v) dextrose, pH 7.4). Platelets were then resuspended in FBS-free medium; for example, platelets from 40 mL of blood in 2 mL medium. To enable platelet activation, PGE1 addition was omitted at this step. Platelets were counted in a Neubauer chamber before the required number of platelets was activated by addition of 0.5 U/mL thrombin (Ref. 1060240000; Roche, Switzerland) for 15 min at 37°C. Activated platelets were used for cultivation with cells and for setting-up invasion assays.

Cell invasion/migration assay

The protocol was performed similar as previously described [1]. Invasion and migration assays were performed in permeable transwell invasion chambers with 8 µm pore size (353097; Corning, New York, US) with 24-well companion plates (353504; Corning, New York, US). Prior to the assay, the cells were sub-cultured either in normal growth medium or with activated platelets (5×10^7 PLTs/ml) for 6-7 days. For invasion assays, the transwell chambers were coated with Matrigel basement membrane matrix (354234; Corning, New York, US). The Matrigel basement membrane matrix (354234; Corning, New York, US) was first diluted in FBS-free medium to 300 µg/mL and then applied to the transwell chambers (100 µL/transwell chamber). This step was omitted for migration assays. After solidification of the Matrigel layer in the CO_2 incubator for 2 h at 37°C, 750 µL complete growth medium were added to each well of the companion plate. At the day of the assay, the cells were harvested and washed with FBS-free growth medium. After live cell counting with trypan blue exclusion staining (TC20 automated cell counter, Bio-Rad Laboratories, California, US), cell suspensions with or without platelets, compounds, and 0.1% DMSO vehicle control, were prepared. 250 µL of each cell suspension were applied to a transwell invasion chamber and incubated in a CO_2 incubator. At day 3, the remaining cells in the upper part of the transwell were carefully removed with a cotton swab. Cells that had migrated and attached to the transwell bottom were fixed with 4% formaldehyde and then stained with crystal violet solution (0.5% in 20% methanol) for 30 min. Subsequently, the transwells were thoroughly washed with water until all residual crystal violet was removed. Microscopic images were taken (Olympus IX50 microscope, Camera Olympus SC30; Olympus, Japan) prior to elution of the crystal violet with 8% acetic acid. Eluates were photometrically quantified at 570 nm with the reference wavelength 690 nm using an absorbance microplate reader (Tecan Sunrise, Tecan, Switzerland). In parallel to the invasion assay, the same experimental setup was seeded in normal tissue culture plates without the transwell chambers to control for toxicity and growth during the assay. The plates were stained and quantified in parallel to the

invasion assay. The ODs of the invaded cells were normalized relative to ODs from tissue culture plates.

Orthotopic xenograft mouse model

The orthotopic xenograft mouse model of Hep3B2.1-7 human hepatic carcinoma was previously established [2]. The experiment was conducted by vivoPharm pty Ltd (Australia) in accordance with the principles outlined in the Australian Code of Practice for the Care and Use of Animals for Scientific Purposes, 7th Edition, 2004 (National Health and Medical Research Council). Procedures involving the care and use of animals in this study were reviewed and approved by the University of Adelaide Animal Ethics Committee. The study was carried out in compliance with the ARRIVE guidelines (http://www.nc3rs.org.uk/page.asp?id=1357).

The animals were delivered to the test facility 11 days prior inoculation. On arrival, all animals received a detailed physical examination, including body weight measurement by the technical staff. All animals were found to be in satisfactory health. Each animal was identified by a transponder (Bar Code Data Systems Pty Ltd, Botany Bay, NSW, Australia) that could be scanned with a barcode reader (DataMars LabMax I). The transponder was implanted by subcutaneous injection between the shoulder blades while the mouse was under isoflurane-induced anaesthesia. The animals were housed in groups of six in micro-isolator cages (two cages per group). Each cage was clearly labelled with a cage card indicating study number, group, gender, and prescribed dose concentration and dose volume. The animals were kept in a controlled environment (targeted ranges: temperature 21 ± 3°C, humidity 30-70%, 15 air changes per hour), with a light/dark cycle each of 12 hours, and under barrier (quarantine) conditions. Temperature and relative humidity were monitored continuously. All animals were subjected to the same environmental conditions. A standard certified commercial rodent diet (Rat and Mouse Cubes, Specialty Feeds Pty Ltd, Glen Forrest, WA, Australia) and tap water were provided to the animals ad libitum. Food supply was sterilized by autoclaving, and water

supply was sterilized by acidification with hydrochloric acid (pH 2.4-2.7) by Laboratory Animal Services, University of Adelaide (Adelaide, SA, Australia). Additional feed (animal seed, Magill Grain Store, Magill, SA, Australia), as well as wet standard feed was provided to all animals from Day 7 onwards, as a measure to minimize body weight loss. The extent of adverse reaction was assessed during the study and mice were culled if severe adverse reaction to the treatment was observed. The age range of the mice were 12-16 weeks at onset of treatment.

Hep3B2.1-7 human hepatic carcinoma cells were sourced from the American Type Culture Collection (ATCC; Virginia, USA). For tumour cell production, Hep3B2.1-7 human hepatic carcinoma cells (passage 4 from working stock) were cultured in RPMI 1640 cell culture medium supplemented with 10% FBS, 1% Glutamax, 1% penicillin-streptomycin and 1% sodium pyruvate. The cells were harvested by trypsinization, washed twice in HBSS, counted, and resuspended in HBSS:Matrigel (34% Matrigel) to a final concentration of 2.5×10^8 cells/mL. Tumours were created by injection of 2.5×10^6 Hep3B2.1-7 cells into the main lobe of the liver of anaesthetized female SCID mice (severe combined immunodeficient, C.B-17-Igh-1^bPrkdc^{scid}). In more detail, while under anaesthesia (via Ketamil (14 mg/mL)/Xylazil (0.9 mg/mL) injection), an incision was made into the skin directly over the liver to expose the main lobe of the liver. The needle was introduced into the main lobe of the liver, where 2.5 x 106 Hep3B2.1-7 cells (in 10 µL with 34% Matrigel™) were discharged. Fifteen days post-inoculation, five mice were culled to assess the size and take rate of tumours. The take-rate of the tumours was 80%. The animals were randomized in treatment groups based on the body weight. Oral treatment with either vehicle control ((NMP:PEG300:Saline (1:9:10, v/v)), resminostat (15 mg/kg and 40 mg/kg), sorafenib (40 mg/kg), or the resminostat/sorafenib combination (15/40 mg/kg, 40/40 mg/kg) commenced at day 0. The Vehicle Control and Test Articles were administered in a dosing volume of 10 mL/kg. Combination therapies were administered in a total dosing volume of 10 mL/kg (5 mL/kg each compound). The dosing volumes were adjusted to the body weight of

the mice. All treatments were administered once daily for 21 days. Body weights were recorded for all animals on the first treatment day (day 0) and then three times per week, including the termination day of the study (Day 21). The treatment of the animals was ceased when the body weight dropped below 85% of that on entry of the study. Mice were euthanized via carbon dioxide inhalation when culled. Individual mice treated with either Vehicle Control (one mouse), 4SC-201 at both doses (15 and 40 mg/kg; respectively; two mice in each group) were found dead or were culled when found with severe lethargy prior to the end of the study. Adverse events and early death of mice in the Vehicle Control and monotherapy groups (4SC-201 at 15 and 40 mg/kg) were considered a result of large tumour size and disease progression. Only mice which were culled on the termination date of the study were used for tumour growth analysis. To determine the tumour weight, livers with intact tumours were removed, the tumours were excised and weighed.

Summary of tumour and body weight of allocated groups:

**Treatments (6 groups)**	**Vehicle control**	**Sorafenib (40 mg/kg)**	**Resminostat (15 mg/kg)**	**Resminostat (40 mg/kg)**	**Resm./Soraf. (15/40 mg/kg)**	**Resm./Soraf. (40/40 mg/kg)**
**Survival**	11/12	12/12	10/12	10/12	12/12	12/12
**Analysed mice per group**	11	12	10	10	12	12
**Average body weight in g at day 0**	22.0	22.0	22.1	22.0	22.0	22.0
**% Delta body weight (rel. Day 0)**	5.2	-5.6	5.6	-0.3	-7.3	-8.6
**Tumour weight (g)**	6.40	1.52	2.61	3.87	0.00	0.00
	4.58	0.24	1.94	2.83	0.00	0.20
	3.45	0.52	0.00	1.65	1.21	0.15
	5.56	1.27	4.26	1.74	1.78	0.36
	0.80	1.11	3.88	0.93	0.36	0.58
	4.92	1.15	0.27	0.21	1.42	0.02
	6.64	0.85	6.45	2.26	1.14	0.03
	7.11	1.78	3.72	0.01	1.48	1.12
	1.98	0.74	2.20	2.81	0.00	0.92
	2.45	2.69	0.16	1.99	0.43	0.45

Treatments (6 groups)	Vehicle control	Sorafenib (40 mg/kg)	Resminostat (15 mg/kg)	Resminostat (40 mg/kg)	Resm./Soraf. (15/40 mg/kg)	Resm./Soraf. (40/40 mg/kg)
	2.94	0.50			0.12	1.99
		0.92			0.17	0.62
**Mean**	**4.26**	**1.11**	**2.55**	**1.83**	**0.68**	**0.54**
**Std. Error of Mean**	**0.63**	**0.19**	**0.66**	**0.38**	**0.19**	**0.17**

Microscopy

Light microscopy was performed with an Olympus IX50 microscope with an Olympus SC30 camera (Olympus). For images depicting cell morphology, 10× magnification was used (10× objective lens). Cells were cultured to 50-70 % confluency prior to microscopy. For cell invasion assay images, the bottom side of the crystal violet stained transwell inserts was photographed at 2× magnification (2× objective lens).

In vitro HDAC inhibition assay

The half maximal HDAC-inhibitory concentrations (IC50) of resminostat were determined by *in vitro* HDAC activity assays (Reaction Biology Corporation RBC, Pennsylvania, USA). For the assay, resminostat was dissolved in DMSO and a dilution series with 10 concentrations was used. The inhibitory activity of resminostat was tested on purified recombinant human HDAC1-11. HDAC1-11 were produced using a baculovirus expression system (human HDAC1: cat# 50051, BPS, California, US; human HDAC2: cat# KDA-21-277, RBC, Pennsylvania, USA; human HDAC3: cat# KDA-22-278, RBC, Pennsylvania, USA; human HDAC4: cat# 50004 BPS, California, US; human HDAC5: cat# KDA-21-280, RBC, Pennsylvania, USA; human HDAC6: cat# H88-30G, SignalChem Lifesciences, Canada; human HDAC7: cat# KDA-21-281, RBC, Pennsylvania, USA; human HDAC8: RBC, cat# KDA-21-481; human HDAC9: BPS, cat# 50009; human HDAC10: BPS, California, US; cat# 50010; human HDAC11: cat# KDA-21-381, RBC, Pennsylvania, USA). The functional activities of HDAC1 to 11 were assessed using acetylated AMC (7-amino-4-methycoumarin)-

labelled fluorogenic peptide substrates: fluorogenic peptide from p53 residues 379-382 (RHKK(Ac)AMC) for HDAC1, 2, 3, 6, 10, 11; fluorogenic HDAC Class IIa Substrate (Trifluoro acetyl Lysine, Ac-LGK(TFA)-AMC) for HDAC 4, 5, 7, 9; and fluorogenic peptide from p53 residues 379-382 (RHK(Ac)K(Ac)AMC) for HDAC8. 1 mg/mL BSA and DMSO was added to the assay buffer (50 mM Tris-HCl, pH 8.0; 137 mM NaCl; 2.7 mM KCl; 1 mM $MgCl_2$) prior to use. In a two-step reaction, the substrate with the acetylated lysine side chain was incubated for with a sample containing HDAC activity to produce the deacetylated products: 1 h at 30°C for HDAC1, 2, 3 and 6; 30 min at RT for HDAC4, 5, 7 and 9; 2 h at 30°C for HDAC8, 10 and 11. In a second step, the deacetylated products were digested by the addition of developer for 1 h at 30°C to produce the fluorescent signal proportional to the amount of deacetylated substrates and the fluorescence was measured using an Envision plate reader at 355 nm (excitation) and 460 nm (emission). A kinetic measurement was performed for ~1 h with 5 min intervals and data from the final time point were analysed. IC50 values and curve fits were obtained using GraphPad Software.

Efficacy screening assay

The experiment was performed by Oncolead GmbH & Co. KG (Karlsfeld, Germany), based on a standardized screening procedure. Cell lines were purchased directly from the ATCC, NCI and DSMZ cell line collections. Cells used for the study had undergone less than 20 passages. To ensure the absence of potential contamination and wrong assignment, all cell lines were tested using a whole genome assay (Agilent, USA). All cell lines were grown as recommended by the suppliers and with addition of 100 U/mL penicillin G and 100 µg/mL; streptomycin. Cell growth and treatments were performed in Nunclon D 96-well plates (NUNC, Germany). The optimal seeding density for each cell line was determined to ensure exponential growth for the duration of the experiment. All cells growing without anti-cancer agents were sub-confluent by the end of the treatment, as determined by visual inspection. After an initial 24 h pre-growth period, the compounds were added. The vehicle concentration

was 0.1% DMSO. After incubation at 37°C for 72 h, the culture medium was aspirated, and the cells were precipitated by the addition of 10% trichloroacetic acid (Sigma-Aldrich, Missouri, US). After overnight incubation at 4°C, the plates were washed three times with deionized water. The precipitates were then stained with SRB (0.4% wt/v sulforhodamine B solution in 1% acetic acid, Sigma-Aldrich, Missouri, US). Afterwards, the plates were washed six times with 1% acetic acid to remove unbound stain. After drying the plates at room temperature, the bound SRB was solubilized with 10 mM Tris base and the optical density was measured at 520 nm using a Victor2 plate reader (Perkin Elmer, Germany). Blank OD values were subtracted from mean OD values for each concentration. The nonlinear curve fitting and EC50 calculations were performed using XLfit and algorithm "205" (ID Business Solutions Ltd., UK)

Statistical analysis

For *cell-based* and other *in vitro* assays, data are presented as data mean ± SD of at least three replicates. The statistical tests that were applied are indicated in the figure legends.

For the orthotopic mouse model, data are presented as mean + SEM. For statistical analysis, one-way ANOVA and multiple comparison (Dunnett) was performed for compound treatments compared to vehicle control. The adjusted p-values with 95% confidence interval were reported as significant. For comparison of EC50 values in Figure 1F, one-way ANOVA with multiple comparison testing (Tukey) was performed. Multiplicity adjusted p-values for the EC50 with sorafenib versus resminostat for each cell line and comparison between cell lines for sorafenib are presented (95% confidence interval for significance testing). For comparison of EC50 data in Figure 2B, one-way ANOVA with multiple comparison testing (Tukey) was performed. Multiplicity adjusted p-values with 95% confidence interval was applied. For the invasion assays in Figure 4, one-way ANOVA with multiple comparison testing (Tukey) was performed. Multiplicity adjusted p-values with 95% confidence interval was applied. The adjusted p-values comparing vehicle control without vs. with platelets and compound

treatments vs. vehicle control in the presence of platelets are presented in the figure. For qPCR analysis, data from three biological replicates are presented as data means with SD. Paired ~~one~~ two tailed t-test was performed.

Supplementary references

1. Labelle M, Begum S, Hynes RO. Direct Signalling between Platelets and Cancer Cells Induces an Epithelial-Mesenchymal-Like Transition and Promotes Metastasis. *Cancer Cell* **2011**;20: 576–90.

2. Yao X, Hu JF, Daniels M, Yien H, Lu H, Sharan H, et al. A novel orthotopic tumour model to study growth factors and oncogenes in hepatocarcinogenesis. *Clin Cancer Res* **2003**;9: 2719–26.

Original western blot scans

Originial scans Figure 5C

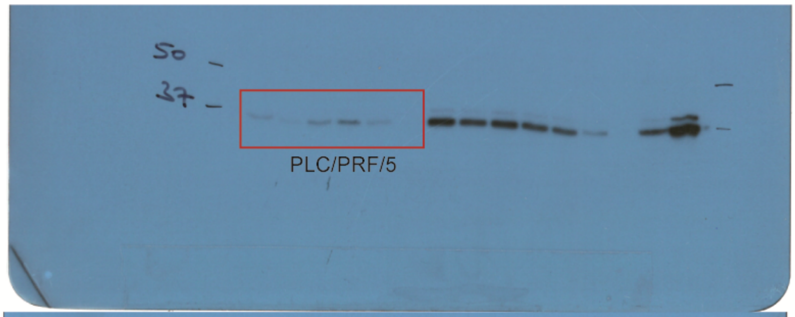

pERK, 1h exposure

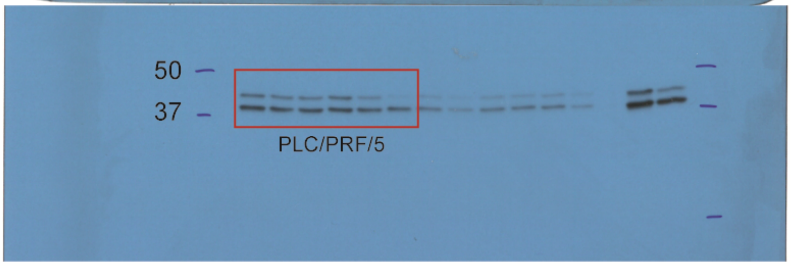

ERK, 10 min exposure

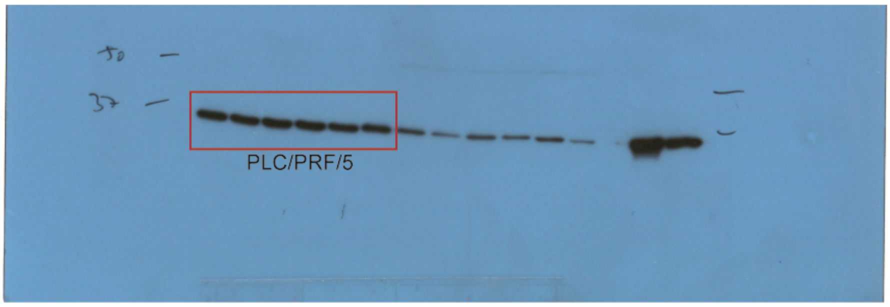

GAPDH, 10 min exposure

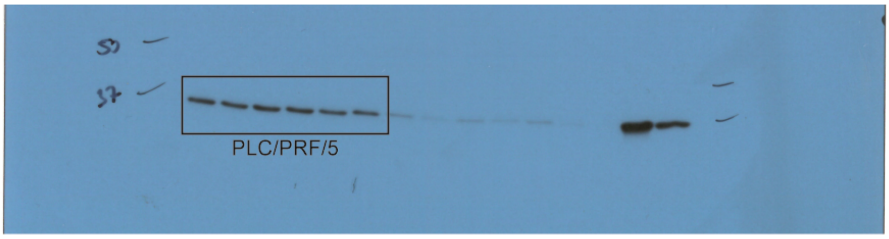

GAPDH, 2 min exposure

Originial scans Figure 5D

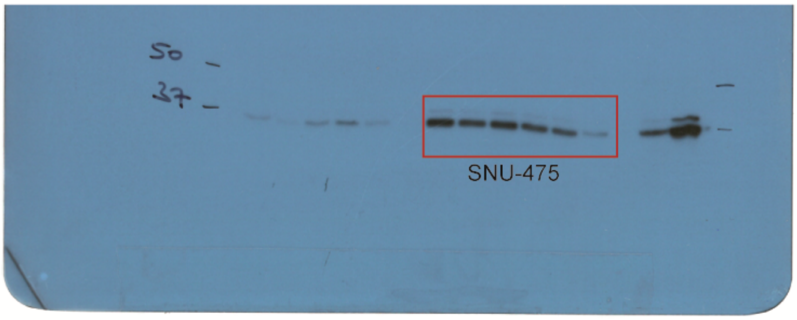

pERK, 1h exposure

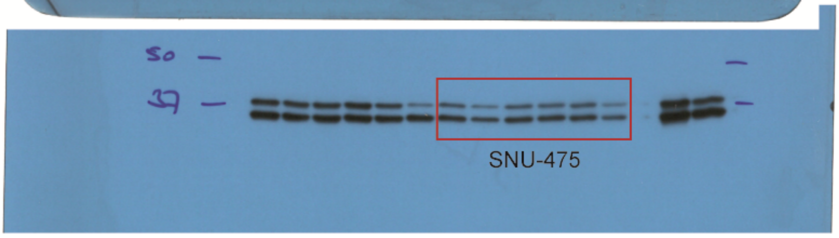

ERK, 50 min exposure

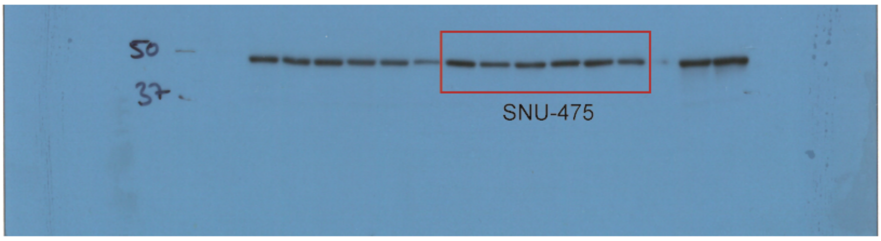

αTubulin, 2 min exposure

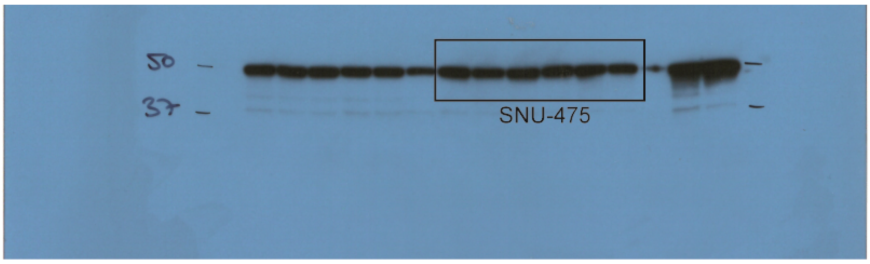

αTubulin, 5 min exposure

Originial scans Figure S1C

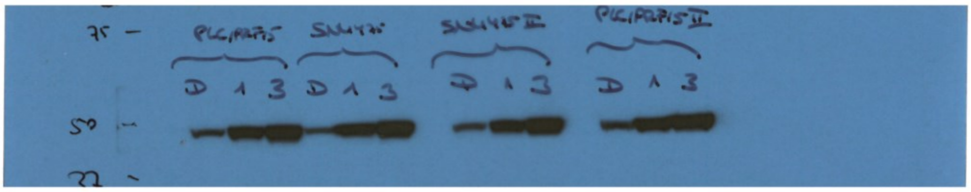

Acetyl-α-Tubulin
30 sec exposure

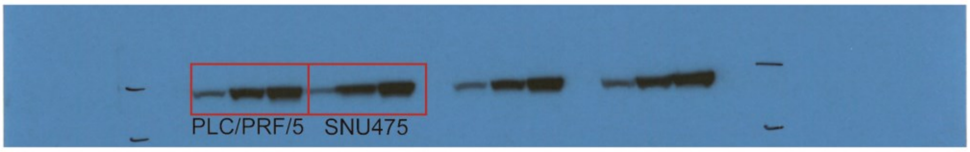

Acetyl-α-Tubulin,
20 sec exposure

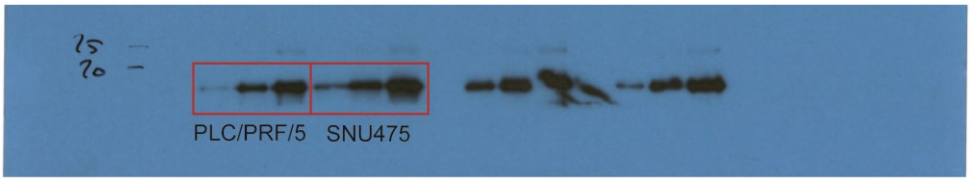

p21,
30 sec exposure

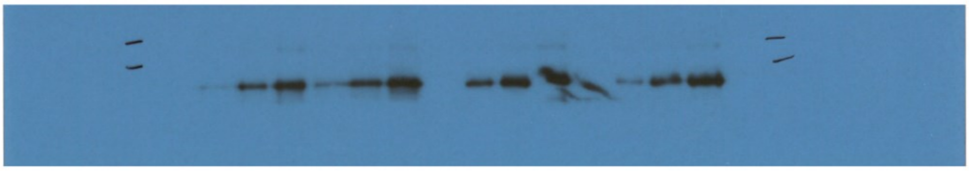

p21,
20 sec exposure

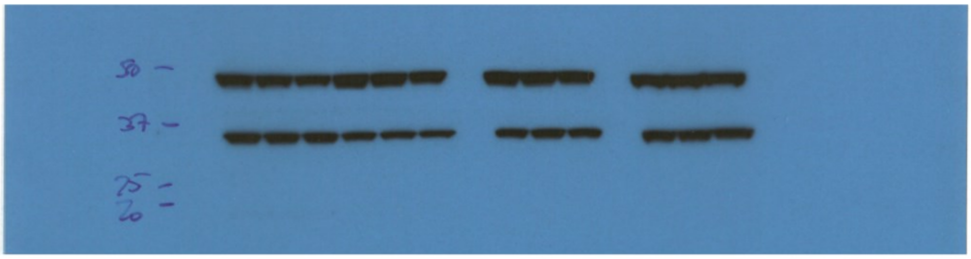

α-Tubulin, 30 sec

GAPDH,
30 sec exposure

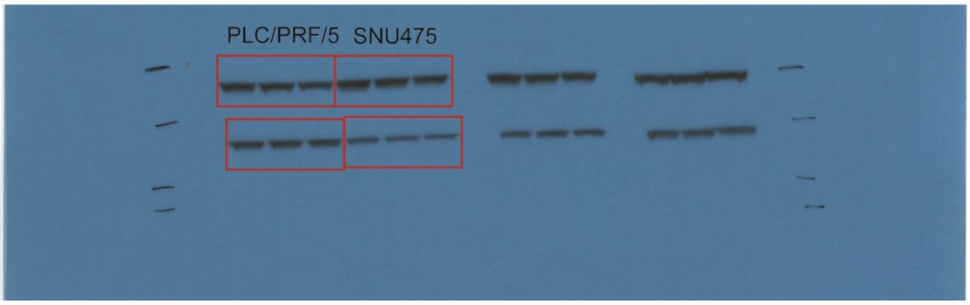

α-Tubulin, 20 sec

GAPDH,
20 sec exposure

The ARRIVE Guidelines Checklist

Animal Research: Reporting In Vivo Experiments

Carol Kilkenny[1], William J Browne[2], Innes C Cuthill[3], Michael Emerson[4] and Douglas G Altman[5]

[1]*The National Centre for the Replacement, Refinement and Reduction of Animals in Research, London, UK,* [2]*School of Veterinary Science, University of Bristol, Bristol, UK,* [3]*School of Biological Sciences, University of Bristol, Bristol, UK,* [4]*National Heart and Lung Institute, Imperial College London, UK,* [5]*Centre for Statistics in Medicine, University of Oxford, Oxford, UK.*

	ITEM	RECOMMENDATION	Section/ Paragraph
Title	1	Provide as accurate and concise a description of the content of the article as possible.	Title
Abstract	2	Provide an accurate summary of the background, research objectives, including details of the species or strain of animal used, key methods, principal findings and conclusions of the study.	Abstract, Result paragraph 3,<br>Materials and Methods see below for details
INTRODUCTION			
Background	3	a. Include sufficient scientific background (including relevant references to previous work) to understand the motivation and context for the study, and explain the experimental approach and rationale.<br>b. Explain how and why the animal species and model being used can address the scientific objectives and, where appropriate, the study's relevance to human biology.	Introduction section<br>Result section → Platelet count dependent impact on drug response to resminostat in combination with sorafenib in an HCC phase I/II clinical trial, paragraph 3
Objectives	4	Clearly describe the primary and any secondary objectives of the study, or specific hypotheses being tested.	Result section → Platelet count dependent impact on drug response to resminostat in combination with sorafenib in an HCC phase I/II clinical trial, paragraph 3
METHODS			
Ethical statement	5	Indicate the nature of the ethical review permissions, relevant licences (e.g. Animal [Scientific Procedures] Act 1986), and national or institutional guidelines for the care and use of animals, that cover the research.	Materials and Methods → Orthotopic xenograft mouse model, paragraph 3

Study design	6	For each experiment, give brief details of the study design including:<br>a. The number of experimental and control groups.<br>b. Any steps taken to minimise the effects of subjective bias when allocating animals to treatment (e.g. randomisation procedure) and when assessing results (e.g. if done, describe who was blinded and when).<br>c. The experimental unit (e.g. a single animal, group or cage of animals).<br>A time-line diagram or flow chart can be useful to illustrate how complex study designs were carried out.	Materials and Methods section: Orthotopic xenograft mouse model, paragraph 3<br><br>Supplementary Materials and Methods → Orthotopic xenograft mouse model, paragraph 7
Experimental procedures	7	For each experiment and each experimental group, including controls, provide precise details of all procedures carried out. For example:<br>a. How (e.g. drug formulation and dose, site and route of administration, anaesthesia and analgesia used [including monitoring], surgical procedure, method of euthanasia). Provide details of any specialist equipment used, including supplier(s).<br>b. When (e.g. time of day).<br>c. Where (e.g. home cage, laboratory, water maze).<br>d. Why (e.g. rationale for choice of specific anaesthetic, route of administration, drug dose used).	Materials and Methods section: Orthotopic xenograft mouse model, paragraph 3<br><br>Supplementary Materials and Methods → Orthotopic xenograft mouse model, paragraph 7
Experimental animals	8	a. Provide details of the animals used, including species, strain, sex, developmental stage (e.g. mean or median age plus age range) and weight (e.g. mean or median weight plus weight range).<br>b. Provide further relevant information such as the source of animals, international strain nomenclature, genetic modification status (e.g. knock-out or transgenic), genotype, health/immune status, drug or test naïve, previous procedures, etc.	Materials and Methods section: Orthotopic xenograft mouse model, paragraph 3<br><br>Supplementary Materials and Methods → Orthotopic xenograft mouse model, paragraph 7

The ARRIVE guidelines. Originally published in *PLoS Biology*, June 2010[1]

Housing and husbandry	9	Provide details of:<br>a. Housing (type of facility e.g. specific pathogen free [SPF]; type of cage or housing; bedding material; number of cage companions; tank shape and material etc. for fish).<br>b. Husbandry conditions (e.g. breeding programme, light/dark cycle, temperature, quality of water etc for fish, type of food, access to food and water, environmental enrichment).<br>c. Welfare-related assessments and interventions that were carried out prior to, during, or after the experiment.	Supplementary Materials and Methods → Orthotopic xenograft mouse model, paragraph 7
Sample size	10	a. Specify the total number of animals used in each experiment, and the number of animals in each experimental group.<br>b. Explain how the number of animals was arrived at. Provide details of any sample size calculation used.<br>c. Indicate the number of independent replications of each experiment, if relevant.	Supplementary Materials and Methods → Orthotopic xenograft mouse model, paragraph 7
Allocating animals to experimental groups	11	a. Give full details of how animals were allocated to experimental groups, including randomisation or matching if done.<br>b. Describe the order in which the animals in the different experimental groups were treated and assessed.	Materials and Methods section → Orthotopic xenograft mouse model, paragraph 3<br><br>Supplementary Materials and Methods → Orthotopic xenograft mouse model, paragraph 7
Experimental outcomes	12	Clearly define the primary and secondary experimental outcomes assessed (e.g. cell death, molecular markers, behavioural changes).	Materials and Methods section: Orthotopic xenograft mouse model, Paragraph 3<br><br>Supplementary Materials and Methods → Orthotopic xenograft mouse model, paragraph 7
Statistical methods	13	a. Provide details of the statistical methods used for each analysis.<br>b. Specify the unit of analysis for each dataset (e.g. single animal, group of animals, single neuron).<br>c. Describe any methods used to assess whether the data met the assumptions of the statistical approach.	Supplementary Materials and Methods → Statistical analysis, paragraph 11
RESULTS			

Baseline data	14	For each experimental group, report relevant characteristics and health status of animals (e.g. weight, microbiological status, and drug or test naïve) prior to treatment or testing. (This information can often be tabulated).	Supplementary Materials and Methods → Orthotopic xenograft mouse model, paragraph 7
Numbers analysed	15	a. Report the number of animals in each group included in each analysis. Report absolute numbers (e.g. 10/20, not 50%[2]).<br>b. If any animals or data were not included in the analysis, explain why.	Supplementary Materials and Methods → Orthotopic xenograft mouse model, paragraph 7<br>Description Figure 1F
Outcomes and estimation	16	Report the results for each analysis carried out, with a measure of precision (e.g. standard error or confidence interval).	Figure 1F<br><br>Supplementary Materials and Methods → Orthotopic xenograft mouse model, paragraph 7
Adverse events	17	a. Give details of all important adverse events in each experimental group.<br>b. Describe any modifications to the experimental protocols made to reduce adverse events.	Supplementary Materials and Methods → Orthotopic xenograft mouse model, paragraph 7
DISCUSSION			
Interpretation/ scientific implications	18	a. Interpret the results, taking into account the study objectives and hypotheses, current theory and other relevant studies in the literature.<br>b. Comment on the study limitations including any potential sources of bias, any limitations of the animal model, and the imprecision associated with the results[2].<br>c. Describe any implications of your experimental methods or findings for the replacement, refinement or reduction (the 3Rs) of the use of animals in research.	Results → Platelet count dependent impact on drug response to resminostat in combination with sorafenib in an HCC phase I/II clinical trial, paragraph 3<br><br>Discussion → Paragraph 2
Generalisability/ translation	19	Comment on whether, and how, the findings of this study are likely to translate to other species or systems, including any relevance to human biology.	Results→ Platelet count dependent impact on drug response to resminostat in combination with sorafenib in an HCC phase I/II clinical trial, paragraph 3<br>Discussion
Funding	20	List all funding sources (including grant number) and the role of the funder(s) in the study.	Funding

References:
1. Kilkenny C, Browne WJ, Cuthill IC, Emerson M, Altman DG (2010) Improving Bioscience Research Reporting: The ARRIVE Guidelines for Reporting Animal Research. *PLoS Biol* 8(6): e1000412. doi:10.1371/journal.pbio.1000412
2. Schulz KF, Altman DG, Moher D, the CONSORT Group (2010) CONSORT 2010 Statement: updated guidelines for reporting parallel group randomised trials. *BMJ* 340:c332.
